# Novel Approaches for the Treatment of Pulmonary Tuberculosis

**DOI:** 10.3390/pharmaceutics12121196

**Published:** 2020-12-10

**Authors:** Zhi Ming Tan, Gui Ping Lai, Manisha Pandey, Teerapol Srichana, Mallikarjuna Rao Pichika, Bapi Gorain, Subrat Kumar Bhattamishra, Hira Choudhury

**Affiliations:** 1 School of Pharmacy, International Medical University, Kuala Lumpur 57000, Malaysia; tan.zhiming@student.imu.edu.my (Z.M.T.); lai.guiping@student.imu.edu.my (G.P.L.); 2 Department of Pharmaceutical Technology, School of Pharmacy, International Medical University, Jalan Jalil Perkasa, Bukit Jalil, Kuala Lumpur 57000, Malaysia; 3 Centre for Bioactive Molecules and Drug Delivery, Institute for Research, Development and Innovation, International Medical University, Kuala Lumpur 57000, Malaysia; mallikarjunarao_pichika@mu.edu.my; 4 Drug Delivery System Excellence Center, Prince of Songkla University, Songkhla 90110, Thailand; teerapol.s@psu.ac.th; 5 Department of Pharmaceutical Technology, Faculty of Pharmaceutical Sciences, Prince of Songkla University, Songkhla 90110, Thailand; 6 Department of Pharmaceutical Chemistry, School of Pharmacy, International Medical University, Kuala Lumpur 57000, Malaysia; 7 School of Pharmacy, Faculty of Health and Medical Sciences, Taylor’s University, Subang Jaya, Selangor 47500, Malaysia; bapi.gorain@taylors.edu.my; 8 Centre for Drug Delivery and Molecular Pharmacology, Faculty of Health and Medical Sciences, Taylor’s University, Subang Jaya, Selangor 47500, Malaysia; 9 Department of Life Science, School of Pharmacy, International Medical University, Jalan Jalil Perkasa, Bukit Jalil, Kuala Lumpur 57000, Malaysia; SubratKumar@imu.edu.my

**Keywords:** tuberculosis, lung physiology, barriers of pulmonary delivery, advanced drug delivery, lung delivery, metered-dose inhaler, proliposome

## Abstract

Tuberculosis (TB) is a contagious airborne disease caused by *Mycobacterium tuberculosis*, which primarily affects human lungs. The progression of drug-susceptible TB to drug-resistant strains, MDR-TB and XDR-TB, has become worldwide challenge in eliminating TB. The limitations of conventional TB treatment including frequent dosing and prolonged treatment, which results in patient’s noncompliance to the treatment because of treatment-related adverse effects. The non-invasive pulmonary drug administration provides the advantages of targeted-site delivery and avoids first-pass metabolism, which reduced the dose requirement and systemic adverse effects of the therapeutics. With the modification of the drugs with advanced carriers, the formulations may possess sustained released property, which helps in reducing the dosing frequency and enhanced patients’ compliances. The dry powder inhaler formulation is easy to handle and storage as it is relatively stable compared to liquids and suspension. This review mainly highlights the aerosolization properties of dry powder inhalable formulations with different anti-TB agents to understand and estimate the deposition manner of the drug in the lungs. Moreover, the safety profile of the novel dry powder inhaler formulations has been discussed. The results of the studies demonstrated that dry powder inhaler formulation has the potential in enhancing treatment efficacy.

## 1. Introduction

Tuberculosis (TB) is one of the major health concerns in the world, with at least 10 million people being infected each year [1]. It is a contagious airborne disease caused by *Mycobacterium tuberculosis,* which is transmitted from person-to-person via the air droplets. *M. tuberculosis* is primarily affecting the lungs causing pulmonary tuberculosis, but it can also adversely affect intestine, bones, joints, and other tissues of the body causing extra-pulmonary tuberculosis. TB can be classified into two groups, which are latent TB and active TB. People with latent TB show asymptomatic and is not transmissible while people with active TB show noticeable symptoms and the disease can spread to others [1,2]. All age groups are at risk of getting affected by TB, however, people with immunodeficiency virus (HIV) infection and suffering from other disease conditions that impair the immune system are at higher risk of developing active TB. According to the World Health Organization (WHO), tuberculosis remains one of the leading causes of death from a single infectious disease agent, including people living with HIV [1]. In 2018, it was estimated globally that 10 million people (5.7 men, 3.2 women, and 1.1 children) were affected by TB with approximately 1.5 million people died from the incidence based on the data reported by WHO [1]. As compared to previous years, there was estimated 1.6% decline in the average TB incidence rate per year from 2000 until 2018, and 2% between 2017 and 2018. The number of TB deaths were reduced by 11% between 2015 and 2018 [1].

One of the greatest obstacles in controlling TB is the emergence of drug-resistant tuberculosis. There are two types of drug-resistant tuberculosis namely: multidrug-resistant tuberculosis (MDR-TB) and extensively drug-resistant TB (XDR-TB). In the case of MDR-TB, the two most potent first-line anti-TB drugs, isoniazid (INH), and rifampicin (RIF) are reported ineffective in treating TB as the TB bacteria are resistant to those drugs. The emergence of MDR-TB is mainly due to the inappropriate use of anti-TB drugs, poor quality of anti-TB drugs, wrong prescription by healthcare providers and premature interruption of the treatment. XDR-TB is a more severe form of drug-resistant TB caused by ineffective management of MDR-TB [3,4]. Additionally, XDR-TB is a form of MDR-TB with additional resistance to any fluoroquinolone and at least one of the three injectable second-line anti-TB drugs (amikacin, kanamycin, or capreomycin). In 2018, there were estimated 484,000 new cases of MDR-TB which was resistant to RIF. About 78% of people developed MDR-TB meanwhile 6.2% of people from MDR-TB had XDR-TB as reported by WHO [1]. The standard TB treatment is to strictly follow six months of drug regimen provided with patient support and supervision. As a simple reason, the inappropriate management of TB and inadequate adherence to the treatment can result in the continuing spread of drug-resistant TB [5].

**Figure 1:**
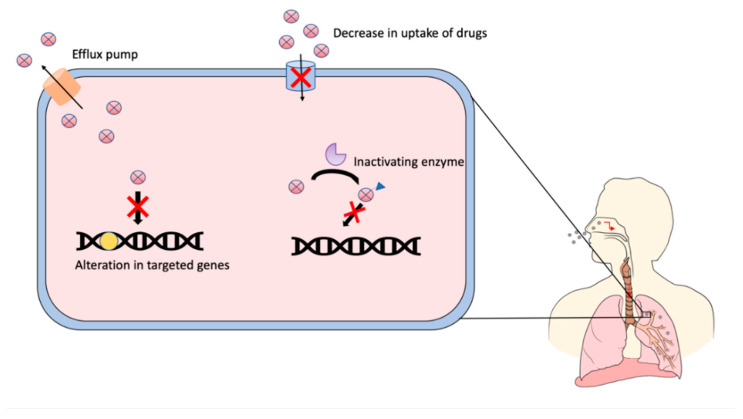
Bacterial antibiotic resistance mechanisms in drug-resistant TB. Mechanisms of bacterial antibiotic resistance include alteration in the drug-targeted genes; decreased permeability of the TB bacteria cell wall which inhibits the entry of antibiotics; degradation of antibiotics by enzymatic action; loss/altered in the drug entry port; expression of efflux pumps on the cell membrane.

Drug-resistant TB is mainly attributed to a mutation in the targeted genes of antibiotics due to previous improper treatment of TB. As a result, the TB bacteria evolve to become more resistant to the prescribed antibiotics [6]. In addition to targeted gene alteration, a decrease in the permeability of the TB bacteria cell wall inhibits the entry of antibiotics into the cell. Consequently, the accumulated antibiotics are degraded slowly by enzymes released from the bacterial cells. Based on the report by Almeida et al., *M. tuberculosis* produces an enzyme called β-lactamase that can degrade β-lactam antibiotics which leads to the resistance of the bacterial towards the respective antibiotics [7]. Besides this, the efflux pump in *M. tuberculosis* can pump the antibiotics out of the bacterial cells which also leads to drug resistance (Figure 1) [8,9]. The treatment for drug-resistant TB requires a longer period to complete and is manageable by second-line anti-TB drugs such as fluoroquinolones, kanamycin, linezolid, bedaquiline, cycloserine and pretomanid. However, the availability of second-line anti-TB drugs is complex and most exhibit several systemic toxic effects such as irreversible ototoxicity, hepatotoxicity, hyperpigmentation and bone marrow toxicity [4,10].

## 2. History of Treatments of TB and Associated Limitations

TB has been called “phthisis” (ancient Greece), “tabes” (ancient Rome), and “schachepheth” (ancient Hebrew) [11,12]. In 17th and 18th centuries, the term “consumption” was commonly used as a lay term for phthisis [12]. In 1834, the term “tuberculosis” was coined by Johann Lukas Schönlein [11]. TB was a lethal disease with no cure until the cause of TB was revealed. The discovery of TB treatment in the 20th century remains one of the greatest achievements in human history. In 1882, Robert Koch had discovered the cause of TB which was the very first step in finding a cure for the disease [11,13,14]. In 1944, Hinshaw and Feldman tested streptomycin, which was only recently discovered by Waksman in 1943, on guinea pigs infected with virulent *M. tuberculosis* [14,15,16]. The guinea pigs tested with streptomycin showed a significant improvement compared to the control group. Streptomycin was then tested on a 21-year-old female, white tuberculosis patient named Patricia on 20 November 1944 [17]. The patient successfully recovered and survived with an active life. Other than streptomycin, oral *para*-aminosalicylic acid (PAS, designed by Lehmann in 1902) was used to treat a Swedish female TB patient named Sigrid on 30 October 1944 and the results were similar to streptomycin [18]. Both drugs showed their effectiveness against tuberculosis, however, the resistant strains of tuberculosis started to appear after either streptomycin or PAS was given as a single agent [14,19,20]. According to the report of the Medical Research Council (MRC) of the United Kingdom, the rate of mortality for patients treated for pulmonary TB with a single anti-TB agent is similar to that of untreated patients [21]. Due to this limitation, combination therapy of streptomycin and PAS was carried out by the MRC, and the results were compared with monotherapy of either PAS or streptomycin [14,19]. The combination therapy showed a marked decrease in the relapse rates and progression of resistant strains [19].

**Figure 2:**
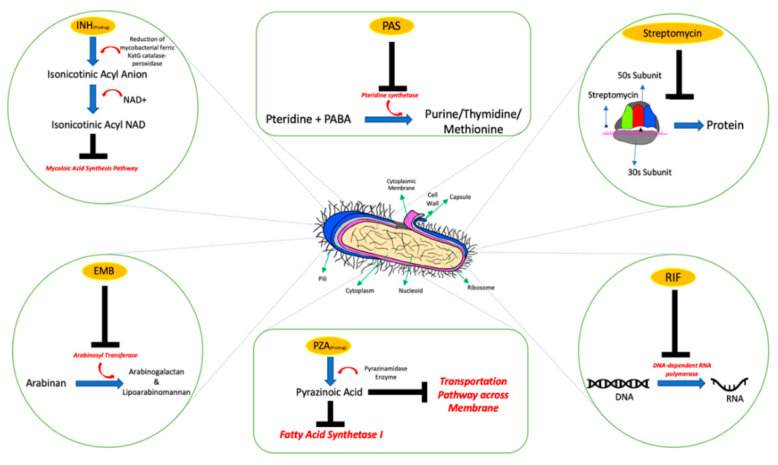
The mechanism of action of anti-TB agents discovered in the 19th century.

INH was first synthesized in 1912 by Meyer and Mally as a part of their doctoral programme in Prague [22,23]. However, the anti-tuberculosis effect of INH remained undiscovered for approximately 40 years until it was independently discovered by the pharmaceutical companies Hoffman La Roche and Farbenfabriken Bayer and the Squibb Institute for Medical Research [14,23]. In 1951, INH, a potent, inexpensive, and safe drug was introduced to treat tuberculosis in clinical trials at the Sea View Hospital in Staten Island [23]. However, INH resulted in resistance in about ¾ of the patients when given as a single agent [14,19]. To overcome this limitation, INH was given with streptomycin and PAS as a combination therapy which as known as ‘triple therapy’. The triple therapy regimen provided an effective effect against bacilli yet the rate of relapse and the number of resistant cases decreased dramatically [19]. In 1934, pyrazinamide (PZA) was chemically synthesized by Dalmer and Walter [24]. Like INH, its anti-tuberculosis activity was not identified until 1952. The discovery depended on the serendipitous observation that nicotinamide possess activity against mycobacteria in animal model [25]. PZA was incorporated with different other anti-tuberculosis drugs to treat TB. The combination of PZA and INH has provided an advantage in the reduction of the treatment course duration, but on the contrary, this combination therapy increased the risks of treatment toxicity. Therefore, only a low dose of PZA can be used as a combination therapy. In 1957 and 1961, the rifamycins and ethambutol (EMB), were discovered, respectively. EMB had been proved effective against streptomycin and INH-resistant strains of tuberculosis via inhibiting arabinosyl transferases of mycobacteria [26]. Rifampint has the advantages of low toxicity, shortened treatment duration (9 months) and enhanced cure rates when combined with INH and EMB. Later on, the clinical trials that conducted by Singapore Tuberculosis Service (STS) and MRC showed that the combination therapy of INH (oral), PZA (oral), and rifampin (oral) has the shortest duration of treatment which is 6 months [14,19]. The mechanism of action of all the anti-tubercular drugs are represented in Figure 2.

Although the combination therapy is effective, the conventional routes of administration (oral and parenteral drug delivery) are associated with adverse effects due to systemic exposure. Furthermore, the invasive nature and short dosing interval of the available anti-tuberculosis agents can also affect the patients’ compliance which results in suboptimal responses or progression of TB to MDR-TB and XDR-TB.

## 3. Anatomy and Physiology of the Lungs

**Figure 3:**
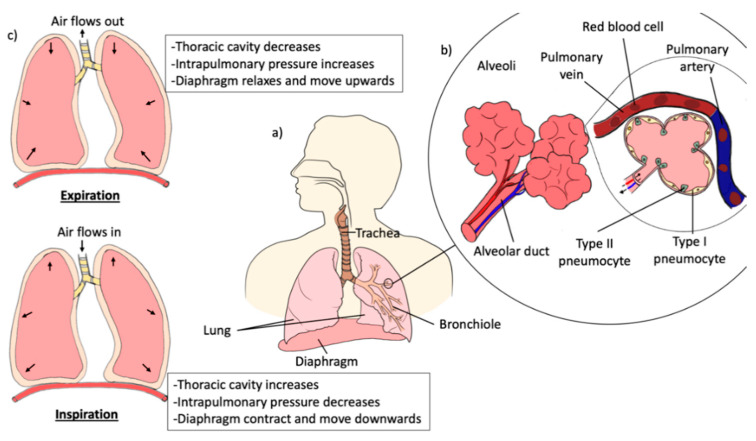
Anatomy and physiology of human lungs. (**a**) Human lungs comprise of trachea, bronchus, bronchiole and alveoli. (**b**) Alveoli is the site where gas exchange occurred. Alveolar epithelium is made up by type I and type II pneumocytes. (**c**) During the inhalation process, the lungs expand and thoracic cavity increases, the intrathoracic pressure is lowered as the lung volume is increased, whereas, during expiration process, the lungs recoil back to its original dimensions, the decreased in volume and thoracic cavity of lungs causes the intrathoracic pressure increased.

To achieve both local and systemic effects, the lungs represent the main organs of the respiratory system which provide the desired route for the direct administration of drugs to the desired site of action. The lungs are cone-shaped, paired organs that are connected to the trachea by the left and right bronchi (Figure 3a). The lungs are located within the thoracic cavity and separated from each other by mediastinal structures. The lungs are surrounded by ribcage, sternum, and spine as protection. The surface of the lungs is covered by two thin protective membranes, the parietal and visceral pleura. There is a space in between the two protective membranes known as the pleural cavity, which consists of lubricating fluid that helps to reduce the friction produced when the two protective membranes slide over each other during respiration [27]. In each lung, the bronchi are branched into smaller bronchioles, which end in tiny air sacs, called alveoli. The alveoli are the functional units of the lungs where gas exchange takes place (Figure 3b) [28,29,30,31]. Alveolar epithelium is made up of type I pneumocytes, which cover more than 90% of the total alveolar surface. Type I pneumocytes are a thin layer squamous epithelium that provides an efficient platform for gas diffusion between the alveolar membrane and the blood capillaries. In addition, alveoli also contain type II pneumocytes which are responsible for surfactant production. Type II pneumocytes also essential for the renewal and repairing of type I pneumocytes. Additionally, type II pneumocytes serve as the reserve cells, which regenerate and replace type I pneumocytes that are damaged, thereby restoring the alveolar structure [31,32,33].

Pulmonary ventilation, also known as breathing, is the process of gas exchange between oxygen and carbon dioxide into and out of the lungs through the respiratory tract. The normal atmospheric pressure at sea level is about 76 mm mercury (mmHg). Therefore, the pressure gradient between the atmospheric and the lungs must be different for respiration to occur. Respiration consists of two major stages: inspiration and expiration. During inspiration, the contraction of the diaphragm and external intercostal muscle causes the ribcage to move outward and upward, which results in the lungs expand and thoracic cavity increases (Figure 3c). The increase in the lung volume results in the intrathoracic pressure lower than the atmospheric pressure, causing the air to flow into the lungs. Alternatively, expiration is a passive process and is dependent on the elasticity of the lungs and the thoracic cage. The external intercostal muscle and the diaphragm relax during expiration, which allows the lungs to recoil back to its original dimensions. The intrathoracic pressure becomes higher than the atmospheric pressure as a result of decrease in the volume of thoracic and lung, causes the air to flow out to the atmosphere. In contrast, deep breathing is an active process as it requires more forceful contractions of the expiratory muscles such as diaphragm, intercostal muscle and the abdominal wall muscles to produce larger changes in the thoracic volume [27,29,34,35,36].

## 4. Different Biological Barriers and Factors Affecting Lung Drug Delivery

### 4.1. Biological Barriers of Lungs

The conventional routes of administration having the disadvantages of first-pass metabolisms and systemic adverse effects respectively [37]. Therefore, pulmonary administration has become one of the most effective, alternative drug delivery routes to treat respiratory diseases especially tuberculosis. Pulmonary drug delivery minimize systemic exposure and increase drug concentration at the targeted site. Thus, the treatment efficacy increases while the systemic side effects can be reduced [38,39]. However, for the drug particles to reach the targeted site, the particles have to be deposited on the luminal surface of the epithelial membrane. Besides, the active ingredients of the drug must be absorbed before being cleared or degraded [40]. There are several biological barriers that need to be overcome by the drug particles before the absorption processes. The biological barriers are similar between the alveolar and airways regions, which are made up of a layer of surfactant/mucus, epithelial layer, basement membrane, and the capillary endothelium. The alveolar regions is the main target site for most lung drug delivery treatment [32].

**Figure 4:**
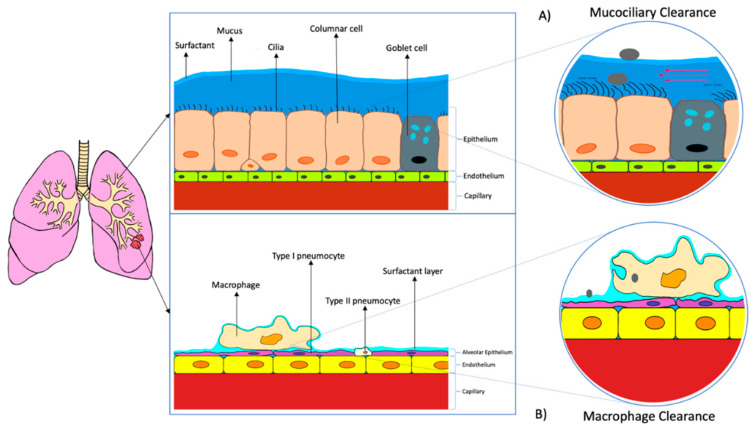
The biological barriers present in the human lungs’ airways and alveolus. (**A**) The pseudostratified columnar cells in airways epithelium possess cilia, which will promote the mucociliary clearance of the inhaled drugs, whereas, (**B**) The macrophages in the surfactant layer of alveolus will carry out macrophage clearance.

The whole barrier at the alveolar region, the air-blood barrier, is the first biological barrier, the surfactant layer. The pulmonary surfactant is located at the inner epithelial surface on the alveoli, which constitutes the advantage of reducing surface tension, which increases the efficiency of gas exchange back and forth the blood capillaries and alveolar region. Therefore, the therapeutic agents should be stable at that surfactant layer (from the attack of enzymes and macrophages), and need to solubilize to cross the barrier if the therapeutics are intended to deliver systemically (Figure 4b). Even though the surfactant layer may act as a barrier to the drug particles, however, the pulmonary surfactant may improve the bioavailability of the drugs through enhanced their solubility [32,40]. Moreover, the mucous layer, which is the first barrier acts as a protective shield against foreign particles. Alternatively, the surface of the airways possess cilia, which is known to increase the clearance of the drugs via mucociliary activity (Figure 4a). Therefore, permeation enhancers can be used to minimize the loss of the drug due to ciliary action [40].

The epithelial cell membrane is the second biological barrier for lung drug delivery. At the alveolar region, the pneumocytes will form a tight alveolar epithelial barrier with high transepithelial electrical resistance. With this barrier, only particles with a size of less than 100 nm can penetrate freely [32]. For the region of the airway, the epithelial cell membrane consists of a layer of pseudostratified columnar cells. The cells are interconnected through tight junctions [40]. To pass through these tight junctions, the drug particles have to be small enough to transport via passive diffusion through the plasma membrane, to transport via specific transporter or able to cross the tight junctions via paracellular transport. If the drug particles are larger, they may require vesicular transport or route to overcome this barrier [32,40].

The last barriers are the basement membrane and capillary endothelium. However, these barriers are not significant for locally acting drugs. They are more significant for the systemically targeting drugs [40].

### 4.2. Factors Affecting Lung Drugs Delivery

Several factors may affect the particle deposition of lung drug delivery. These factors are categorized into 2 types which are physiological factors and pharmaceutical factors [41].

#### 4.2.1. Physiological Factors

(a) Lung morphology: The architecture of the lungs’ airways can affect the efficiency of pulmonary drug delivery as the diameter of the airways decrease from the trachea to bronchioles. The risk of impaction increases for every bifurcation/branching which will decrease the efficiency in pulmonary drug delivery into deep lungs [42,43]. Furthermore, the airflow in the airways will be influenced and results in the generation of turbulent (due to the sudden decrease in the diameter of airways). The formation of turbulent will increase the deposition of the drug particles on the upper airways [44].
(b) Inspiration flow rate: When the airflow rate is fast, the drug particles are more likely to deposit on the upper airways and oropharynx. In contrast, a slow/moderate airflow will reduce the momentum and the possibility of impaction. Therefore, more particles can be travel to the lower respiratory tract. Research showed that there are fewer drug particles deposited in deep lungs for the inspiratory flow rate at 60 L/min compared to at 15 L/min due to the strong turbulent dispersion [45,46].
(c) Breath-holding: Breath-holding can prolong the residence time of the drug particles in the respiratory tract to allow the occurrence of sedimentation and increase the lung dose [42]. For instance, Horváth et al., demonstrated that the lung dose increased by a mean value of 21.4 percent (5 s breath-hold) and 42.4 percent (25 s breath-hold) compared to no breath-holding [47].
(d) Disease state: Most of the respiratory diseases such as cystic fibrosis, asthma and chronic bronchitis will cause the narrowing of airways and the bronchial obstruction due to the inflammation and excessive production of mucus [44,48] The change in the diameter of airways will result in the change in airflow velocities, turbulent, and air resistance. As a result, a higher amount of drug particles will deposit in the upper respiratory tract rather than deep lungs [44].

#### 4.2.2. Pharmaceutical Factors

(a) Aerosol performance: The aerosol performance of a dispersion device is significant to lung drug delivery. For example, the aerodynamic diameter of 1–5 µm, fine particle fraction > 35%, and emitted dose of > 90% are the 3 important criteria for good aerosol performance for dry powder inhaler [49,50]. In addition, the airflow velocities of a dispersion device can also influence the lung drug delivery as the greater the velocities of the airflow, the drug particles are more likely to impact in the oropharyngeal area [42,46].
(b) Particle shape/morphology: The shape of the drug particles can influence the particle adhesion in lungs. For instance, researchers showed that the particles with pollen or needles shape enhanced the drug deposition in smaller airways which increased the effectiveness of lung drug deposition [51,52]. Hassan et al., also reported that the pollen shape particles possess the benefits of higher fine particle fraction and minimized drug loss [51].
(c) Physical stability: The physical stability of drug particles is extremely important because the drug particles need to go through several environments with high humidity before reaching the targeted site. Premature deposition may occur if the drug particles are unstable [42]. The physical instability of the drug particles may also lead to loss of encapsulated active pharmaceutical ingredients as well as the aggregation or sedimentation of drugs during storage [50]. Moreover, the physical stability of inhalers is also significant in ensuring the delivery of an equivalent and sufficient dose over the shelf life [53].

## 5. Novel Approaches for Dry Powder Inhalable Lung Drug Delivery

The conventional anti-tuberculosis treatments are effective in most of the cases but require long-term treatment, precise dosing and frequencies, thereby reducing patient compliance. The associated systemic toxicity of the first, second and third-line anti-TB drugs during oral or parenteral delivery also often leads to patient adherence issues, which further contributes to the emergence of serious drug-resistant TB [54,55]. Oral administration has the disadvantages of slow onset of action, undergoing first-pass metabolism, low targeted site concentration and high systemic exposure, whereas intravenous administrations are invasive and often lead to treatment-related toxicity due to high systemic exposure. Compared to oral and intravenous administrations, pulmonary drug delivery possesses numerous advantages such as better in targeting alveolar macrophages with deeper lung deposition, avoid first-pass metabolism, larger surface area for absorption, fast onset of action, non-invasive therapy which promote self-administration. Moreover, pulmonary delivery systems have great potential in improving the therapeutic effects as it delivers drugs directly to the lungs which result in higher drug concentration at the targeted site with lower systemic toxicities and frequency of dosing is reduced [56,57]. In addition to pulmonary drug delivery, micro or nanotechnologies are often incorporated into anti-TB treatment to increase the treatment efficacy. Furthermore, some of the novel formulations possess sustained drug release properties which have the potential in reducing systemic toxicity, dose and frequency of the therapy, hence, lead to the enhancement in treatment efficacy. Among other inhalable formulations such as nebulizer and pressurized metered-dose inhalers, inhalable dry powders formulation has been widely used due to its better physiochemical stability compared to liquid or suspension-based formulations. Furthermore, dry powder inhalers (DPIs) are portable, easy to handle, and the inhaler devices are affordable [58,59]. In this review, the novel approaches in DPI formulation with anti-TB drugs and vaccines for the treatment of TB are discussed in the following sections.

### 5.1. Approaches for Pyrazinamide (PZA) Delivery’

PZA is an antibiotic, which specifically targets *M. tuberculosis*. It has been proven effective against both drug-susceptible and drug-resistant strains of TB [60]. PZA is usually given with other first-line anti-TB agents, such as INH, ethambutol and RIF, as a combination therapy to enhance the treatment efficacy and to reduce the duration of the anti-TB treatment course. However, the conventional use of PZA is associated with several systemic adverse effects, for instance, malaise, nausea and vomiting, anorexia, arthralgia and myalgia. These have opened up the opportunities in developing PZA in an inhalable form dry nanopowder to decrease the systemic exposure while increasing the targeted site concentration.

In 2014, Kaewjan and Srichana developed nano spray-dried PZA-l-leucine dry powder by using ethanolic solvent. Hollow shape particles were formulated due to the surface activity of l-leucine, which caused l-leucine to accumulate at the air-water interface of droplet and inhibited the solvent vapor to penetrate during the spray drying process. The hollow shape particle morphology is significant in reducing the particle density, thereby, decrease the tapped density [61]. Other than that, the dimensions of the hollow particles are larger when higher concentration of ethanol is used. However, the concentration of ethanol did not have direct impact on aerosolization performance. The addition of leucine enhanced the spray-dried nanopowder yield in a range of 31.9–34.4% in comparison to spray-drying pure PZA. The spray-dried yield indicates the aerosolization properties of the formulation which a lower yield demonstrates the particles of the formulation have higher degree of cohesiveness, hence, reduced the aerosolization properties [62]. The overall percentage of drugs emitted from the inhaler device, which as known as emitted fraction (EF), was in high percentage, which was ranged from 86.5–98.4%. Furthermore, PZA was proven stable during the process of spray-drying as the drug content/drug loading for both pure PZA and PZA-leucine formulations were in the acceptable range of 83.1–99.7%. This study showed that co-spray dried of PZA with 20% l-leucine in 10% of ethanol provided the best aerosolization performance among other formulations which provided a fine particles fraction (FPF) of 33.0 ± 4.1% and mass median aerodynamic diameter (MMAD) of 2.83 ± 0.04 µm. FPF represents the proportion of the particles that fall within the respirable diameter of <4.4 µm while MMAD defines as the diameter of the particles at which 50% of the drug mass fall below the stated diameter [63,64]. Other than FPF and MMAD, geometric standard deviation (GSD) value is one of the important parameters in developing dry powder inhaler with good aerosolization properties, which represents the distribution of the particle size, the lower the GSD value, the narrow the distribution of the particle size. The aerosol is considered polydisperse if the GSD value is ≥1.22 and the GSD value of most therapeutic aerosols is in a range of 2 to 3 [65]. Overall, the co-spray-dried PZA with l-leucine increased the FPF by around 2-fold, providing, at the same time, the advantages of reducing MMAD and GSD values compared to spray dried pure PZA. The loss of α-polymorph peaks and the formation of new γ-polymorph peaks were observed. Therefore, the co-spray-dried PZA with optimum amount of l-leucine via nanospray drying technique is one of the approaches for PZA-DPI development [66].

Pham et al., formulated PZA-loaded large porous particles (PZA-LPPs) for the pulmonary delivery route in 2015. The PZA-porous particles are composed of PZA, 1,2-dipalmitoyl-sn-glycero-3-phosphatidylcholine (DPPC), hyaluronic acid (HA), ammonium bicarbonate and leucine combined via a spray drying method. The addition of leucine and ammonium bicarbonate leads to the formation of spherical-shaped particles with lowered tapped density compared to spray-drying pure PZA, while, HA and DPPC play an important role in the formation of stable partially crystalline spherical particles. Spherical shape PZA-LPPs with good aerodynamic properties were determined through a DPI (Aerolizer^®^, Novartis, Basel, Switzerland). The formulation possesses a high EF which is 99 ± 3% and a spray-dried yield of 47.3 ± 0.8%. Furthermore, the drug content for the formulation was 34.9 ± 2.2%. The MMAD of the PZA-LPPs was 4.1 ± 0.2 µm with FPF of 40.1 ± 1.0%. Moreover, the alveolar fraction (AF) and GSD were 29.6 ± 3.1% and 2.16 ± 0.16 respectively. For the in vivo pharmacokinetic studies carried out on male Sprague Dawley rats, the results showed that PZA-LPPs via intratracheal insufflation increased the PZA concentration in epithelial lining fluid (ELF) by 28% compared to intravenous administration. In addition, the PZA concentration in ELF was 2 times higher than the concentration in plasma. Further investigations is required on the infected animals to evaluate the efficacy of PZA-LPPs against *M. tuberculosis* [67].

Other than PZA-LPPs, DPPC can also be incorporated with other excipients to form different types of nanocarrier, which possess the ability to enhance the aerosolization properties of the drugs. In 2016, Eedara et al. developed phospholipid-based PZA inhalable powders by spray-drying method. The powder particles were made up of PZA, DPPC, N-(carbonyl-methoxy polyethylene glycol-2000) (DSPE-PEG2k) and l-leucine. The process yield of spray-dried PZA without excipient was 20%, while the yield of PZA with different fraction of excipients increased significantly to a range of 39–45%. This is because the phospholipid and l-leucine reduce the adhesion force of the particles to the cyclone wall which results in the increased yield at the collecting chamber [68]. Besides, the moisture content of the formulations was lowered than 2% which were within the acceptable range of below 4–5%. The low moisture content increased the dispersion performance of the DPI by reducing the formation of inter-particulate capillary forces [69,70]. In addition, the tapped density of phospholipid-based PZA was smaller than the spray-dried pure PZA which indicated the enhancement in aerosol performance of the DPI [71]. The particles composed of phospholipid and l-leucine tend to form smaller porous, hollow particles which play an essential role in reducing particle density. The decrease in particle density increases the ability for the particles to travel into deep lungs due to the decrease in particle aerodynamic diameter. The in vitro aerosol dispersion performance was tested via Next Generation Impactor™. The emitted dose (ED) for formulations was >70%. Additionally, the results showed that FPF of the phospholipid-based increased significantly compared to pure PZA powder. The pure PZA powders only possess the FPF of 8.5 ± 0.98% whereas the highest FPF from the phospholipid-based PZA formulation (formulation with 25% DPPC) was 73.2 ± 4.0%. The results showed that the aerosolization properties (FPF and GSD) of the phospholipid-based formulation improved significantly with increasing fraction of DPPC. Thus, the formulation with 25% DPPC was the best formulation among other formulation with different DPPC fractions which obtained the MMAD of 2.5 µm and GSD values of 1.75 ± 0.1. These results indicated that this formulation is suitable to delivery into deep lungs. Lastly, the transformation of α-polymorphic crystalline drug to γ-polymorphic form during the spray drying process was confirmed via the solid-state characterization. The phospholipid-based PZA DPI formulation is appropriate to use in pulmonary delivery system, however, evaluation of therapeutic potential is necessary for the future [72].

Similarly, another form of free-flowing dry powder forms, proliposomes, has been introduced into the research, which upon hydration form the configuration of liposomal carriers [50]. This dry-powder delivery approach could overcome the limitations related to aqueous formulation of liposome. With this concept, Rojanarat et al. had formulated inhalable PZA proliposomes dry powder for targeting alveolar macrophages in 2012. The PZA-proliposomes were made up of PZA, L-α soybean phosphatidylcholine (SPC), porous mannitol and cholesterol. Porous mannitol was used as the carriers of PZA due to its low density compared to non-porous particles. This study suggested that the content of porous mannitol should be >60% to form the perfect spheres shape particles. In contrary, the formulations with low porous mannitol content will lead to the formation of irregular shape particles that influence the aerosolization properties. All the PZA-proliposomes formulations (differing in porous mannitol content) showed the MMAD of 4.26–4.39 µm and the FPF were in a range of 20 to 30%. FPF of the formulation decreased when the content porous mannitol decreased from 90 to 60%. However, the FPF increased slightly when the porous mannitol content was further reduced to 40% and 20% which may be resulted from the formation of lower size particles during the spray drying process. The ED of all the formulations were larger than 80%. Noteworthy, the formulation with 90% of porous mannitol possessed the highest FPF and ED which were 29.0 ± 4.1% and 97.8 ± 1.0% respectively. Besides, the results reported that the encapsulation efficiency (EE) of the increased when the PZA:porous mannitol ratio decreased. Thus, the formulation with 1:9 of PZA:porous mannitol ratio exhibited the highest EE among other formulations which was 44.6 ± 0.5%. Besides, the PZA-proliposomes formulation with 90% porous mannitol can be taken up by alveolar macrophages effectively as the vesicle size after reconstitution was 200 nm which are similar to the size of typical nanovesicles. For the cytotoxicity test of PZA-proliposomes to the cells in the respiratory tract, the cell viability of human bronchial cells (Calu-3), human lung adenocarcinoma cells (A549) and alveolar macrophages (AMs) (NR8383) after being exposed to PZA-proliposomes formulation were higher than after being exposed to PZA which suggested that proliposomes formulation is effective in reducing the drug toxicity to the respiratory cells. The study also showed that PZA-proliposomes formulation did not activate AMs to release inflammatory mediators. Generally, the results of this study suggested that PZA-proliposomes formulation is a potential candidate for the treatment of pulmonary TB [73].

### 5.2. Delivery Approaches of Isoniazid

INH is one of the first-line antituberculosis drugs administered via an oral route. However, the conventional delivery system of INH possesses the drawback of poor cell targeting which might increase the possibility of the occurrence of adverse effects which in turn decreases the patient’s compliance and leads to the progression of MDR-TB and XDR-TB [74]. Therefore, INH dry powder inhaler via pulmonary route is a worth trying novel approach that has the advantage of site-targeted delivery or specific cell-targeted delivery.

In 2011, Rojanarat et al. developed an INH-proliposomes dry powder formulation for pulmonary administration. The composition of the INH-proliposomes formulation was similar with PZA-proliposomes formulation, except for the active ingredient and mannitol was used instead of porous mannitol. However, different from the PZA-proliposomes, the spherical mannitol microparticles only observed in the formulation with mannitol content of higher than 90%. When the mannitol content of INH-proliposomes was 80% or lowered than 80%, irregular shape particles will be formed. This was due to the spray drying process which will change the morphology and crystallinity of the formulation. Furthermore, the content uniformity of all the INH-proliposomes formulations with different mannitol content showed uniform distribution. However, the loss of INH was noticed when the INH content is lowered than the mannitol content. This is because the low content INH may exist in an amorphous state which leads to the melting or deposition of the drug on drying chamber during the spray drying process. The mean vesicle size range for the INH-proliposomes formulations after reconstitution was between 300 to 1000 nm. Although the mean size range was higher than the size for AMs to uptake efficiently (200 nm). However, the formulations with mean vesicle size range of 300–400 nm were in fact have the vesicle size distribution between 100 nm to 500 nm. Thus, AMs are still able to uptake some of the vesicles, while the larger particles can play a role in extracellular killing. Besides, the study also showed that the formulations with low INH loading will have higher EE as more INH can be incorporated into porous mannitol particles before lipid coating. The MMAD of all the proliposomes formulation was below 5 µm, but the FPF obtained were relatively low compared to the PZA-proliposomes formulation due to agglomeration of the particles. Therefore, porous mannitol is a more appropriate choice instead of smooth-surface particles. The INH-proliposomes showed nontoxic to normal human bronchial cells (NHBE), small airway epithelial cells (SAEC) and AMs and did not activate AMs to release of inflammatory mediator. Other than that, INH-proliposomes demonstrated a better antimycobacterial activity compared to free INH against *M. bovis*-infected AMs. This was due to the increase in permeation ability of INH against AMs when incorporated into proliposomes and lead to an increase of INH intracellular concentration. The study proved that INH-proliposomes are potential options for alternative TB treatment [50].

Other than proliposomes, polymeric drug-loaded microspheres are also a novel approach which have the potential in improving the treatment efficacy. Kundawala et al., had formulated INH-loaded polymeric microspheres for pulmonary delivery by spray drying method. The drug-loaded microspheres were composed of INH, chitosan, tripolyphosphate (TPP), leucine and lactose. The degree of deacetylation is an important parameter that can influence the interaction of chitosan with the crosslinkers and media. A high degree of deacetylation of 89% was obtained in the chitosan used which indicated that it has a good solubility in water [75,76].

Chitosan is a widely used natural polymer [77], which can also be used in developing nanoparticles other than microspheres. Pourshahab et al., developed inhalable powders of INH containing chitosan polymeric nanoparticles via spray-drying method. Similar to the previous studies, this study emphasized that the ratio of chitosan:TPP was important to the EE, particle size and the drug release profile. The previous study had focused on the effect of concentration of TPP on chitosan particles while this study had further explained the effect of l-leucine and lactose in enhancing the aerosolization properties of the powders. The process yield showed an increase of 10–20% in the formulations with leucine compared to without leucine due to the anti-adherent property of leucine [61]. Furthermore, the formulation with lactose as the excipient possesses the highest process yield compared to the formulation with mannitol or maltodextrin as the excipient. The low process yield in the formulation with mannitol or maltodextrin was caused by the adherence of powders to the inner surface of spray dryer cyclone due to the its stickiness. Besides, leucine was found to have certain effects on the shape and morphology of mannitol and lactose-containing particles. The addition of leucine transformed the irregular (mannitol) and doughnut (lactose) shaped particles into spherical particles with rough surfaces. This dispersibility enhancing property of leucine improved the aerosolization properties of the INH-loaded nanoparticles. For instance, the FPF for the formulations with leucine is significantly higher than the formulations without leucine. For the formulations without leucine, the formulation with lactose obtained the highest FPF while the formulation with maltodextrin showed the lowest FPF. The low FPF in the formulation with maltodextrin may due to the adhesion of powders to the inhalers, hence a decrease the ED and leading to the decrease in FPF. For the microbial study, the results showed that the minimum inhibitory concentration (MIC) of INH solution against *M. avium* was 16 times higher than the INH-loaded chitosan-TPP nanoparticles. Interestingly, the nanoparticles without drugs possess little mycobacterial effects. This may result from the adhesion of positively charged nanoparticles to the negatively charged bacterial cell surface which interferes with the transport of electrons or blocks the intracellular transportations. Overall, l-leucine showed significant effects on the aerosolization properties of the spray-dried powders consisted of drug nanoparticles and the excipients [78].

Oliveira et al., further interpreted the importance of using low molecular weight polymer to avoid toxicity to alveolar macrophage and as toxicity problems had been reported on medium molecular weight chitosan microparticles (190–310 kDa) to murine macrophage cell lines (J-774.1 cells). They developed INH-microparticles with low molecular weight chitosan (50–190 kDa) by using a spray-drying method. Low production efficiency of microparticles in the range between 30.5 to 46.3% was obtained, which was comparable to previous studies [79]. This resulted from the formation of chitosan films on the wall of the spray drying equipment during the spray drying process [80]. Furthermore, the mean particle size of the microparticles ranged from 3.2–3.9 µm which was suitable for pulmonary delivery. Generally, INH-loaded chitosan microparticles possess smooth, spherical shapes. However, the chitosan microparticles without INH presented an irregular, rough appearance. These results showed that the drug-polymer interactions have a certain influence on the particle morphology. For the drug release study, the non-cross-linked chitosan microparticles released loaded INH at a higher speed compared to the dissolution of free INH which the non-cross-linked chitosan microparticles released 82% of INH and 45% of free INH dissolution at the first 90 min [79]. The increase in the drug dissolution was due to the conversion of INH crystalline form to amorphous form during the spray drying process [81,82]. Again, with the addition of TPP into the chitosan microparticles, the drug was released in a sustained release pattern. Besides, the mucoadhesiveness of the chitosan microparticles to the mucous in respiratory tract was also investigated in this study. All the chitosan microparticles showed positive zeta potential between 17.7 mV and 29.8 mV at the beginning and then decreased (zeta potential remained positive) when incubated in mucin dispersion [79]. These results proved the formation of ionic bond between chitosan and mucin was presence which indicated that chitosan microparticles were mucoadhesive [83]. Due to this mucoadhesive property, the concentration of INH in mucosa treated with INH-loaded chitosan microparticles was found to be 6-fold higher than the mucosa treated with free INH as mucoadhesive property able to reduce the expulsion of INH with expiration. The cytotoxicity test on alveolar murine (AMJ2-C11) and J774.1 macrophages cell lines demonstrated that low molecular weight chitosan microparticles did not show cytotoxic activity to the macrophage cell linage. In summary, low molecular weight chitosan microparticles are promising carriers for INH pulmonary administration [79].

Bhardwaj et al., had formulated INH lipid-polymer hybrid nanoparticles (LPN) for pulmonary delivery via spray drying method. The INH lipid-polymer hybrid nanoparticles were composed of INH, soy lecithin, DSPA-PEG2K, poly(lactide-co-glycolide) (PLGA) and mannitol. The INH-LPNs were found to display spherical shapes with smooth surfaces. The particle size of INH-LPNs was 111.81 ± 1.2 nm with a polydispersity index (PDI) of 0.189 ± 1.4, which indicated that INH-LPNs can be efficiently uptake by AMs and the size distribution was narrow. Again, the INH-LPNs formulation was proven to have higher uptake efficiency by J774A.1 cells compared to free INH in in vitro uptake study. In addition, the drug entrapment of INH-LPNs was found to be 63.64 ± 2.12% which showed that the developed LPNs possess a good loading capacity against INH. INH-LPNs obtained a low tapped density of 0.13 ± 0.011 g/mL and an angle of repose of 24.2 ± 2.13°. This demonstrated that INH-LPNs have good aerodynamic properties and excellent flowability respectively. Other than that, the MMAD of 2.49 ± 0.12 µm and FPF of 64.1 ± 1.2% showed that INH-LPNs have good aerosolization properties, which are able to reach into the lower respiratory region. The ED of the INH-LPNs was 88.34 ± 1.42%. For the in vitro drug release study, an initial burst release of INH was observed, followed by a constant release of INH. INH was released from LPNs in a controlled and sustained manner via diffusion and desorption at pH 5.2 (macrophage pH) release media after the initial burst. The sustained drug release properties of INH-LPNs were further confirmed by the in vivo study performed on mice using dry powder insufflators which INH was still detectable in the plasma of mice after 24 h of administration of INH-LPNs compared to free INH (not detectable after 24 h). The INH-LPN formulation is a potential approach for pulmonary delivery in the treatment of TB [84].

### 5.3. Delivery Approaches of Ethambutol

EMB is a bacteriostatic drug that interferes with the biosynthesis of arabinogalactan in the bacteria cell wall, stopping the bacilli from multiplying [85]. EMB belongs to one of the first-line regimens for tuberculosis treatment and acts as protection against unrecognized resistance to one of the three core anti-TB drugs [86]. It has been growing interest in formulating novel dosage forms that deliver EMB directly to the targeted tissue due to the associated systemic adverse effects of conventional treatment such as ocular toxicity, hepatoxicity, thrombocytopenia and neuropathy during long-term treatment.

In the study done by Elham et al., EMB-loaded solid lipid nanoparticles (SLNs) using DPI platform were developed. SLNs are colloidal drug carrier composed of physiological lipid that is biodegradable and biocompatible with advantages of low toxicity, good stability and having controlled drug released profile [87,88,89]. EMB-loaded SLNs were prepared via homogenization followed by ultrasonication with various concentrations of EMB. SLNs are very small in particle size (<1 µm) where the particles often being exhaled without deposited at the lungs, thereby, spray drying method was carried out to formulate SLN particles into optimal size of 1–5 µm for dry powder inhaler form [90]. SLN loaded with 300 mg of EMB showed the best formulation for DPI preparation as its EE was high with 99.04%, PDI was 0.253, drug loading (DL) was 29.71% and particle sizes were below 60nm. EMB loaded SLN microparticles were spray dried with and without mannitol, the results showed that the particle size was 1.12 µm and 1.15 µm respectively, which were within the range for deep lung deposition. EMB-loaded SLN was shown to have good physical stability at low temperature where the formulation was kept at temperature 4 °C for 4 weeks showed no significant change in PDI, particle size, percentage of EE and percentage of DL during this period. Furthermore, the release rate of free EMB and EMB-loaded SLNs was compared and showed 40% and 25% in the first 7 h respectively, which indicates the drug release from SLN was controlled. The controlled release profile of SLN formulation can be used to conquer the effects of high dose needed in conventional treatment and reduce the unwanted effects of the drug. Mannitol was added to the EMB-loaded SLN to improve the flowability of the dry powder. Dry powder with good flowability must have a Carr’s index of lower than 25% and Hausner ratio lower than 1.25. The results of this study showed that the Carr’s index and Hausner ratio of EMB-loaded SLN were 6.43 and 1.068 respectively, which were lower than mannitol-free dry powder. This demonstrated that ethambutol-loaded SLN possesses better flowability due to its spherical shape observed in the developed formulation [91]. The authors concluded that development of ethambutol-loaded SLN in the form of DPI has a great potential for tuberculosis treatment with controlled and better stability during storage. 

Additionally, Ahmad et al. attempted in 2014 another approach where they loaded ethambutol dihydrochloride (EDH) using chitosan DPI formulation to improve the treatment outcome with lower dose required. In the study, EDH DPI formulation was prepared by nanospray drying method with chitosan as the drug carrier. Different nanoparticles were developed with various concentrations of chitosan where they kept EDH concentration fixed with formulations of 1:2, 1:2.5, 1:3.3, 1:5 and 1:10. All the EDH-chitosan DPI formulations produced have good aerodynamic behaviour, with MMAD of 2.3 to 2.9 µm as the chitosan concentration increases whereas the percentage of FPF for all the formulations developed was decreased from 42 to 31% when the chitosan concentration increased. In addition, chitosan acts as a permeation enhancer as EDH DPI with 1:5 and 1:10 formulations had a minimum inhibitory concentration (MIC) of <1 µg/mL whereas pure EDH and chitosan had a MIC at 2 µg/mL and 4 µg/mL respectively. This can be attributed to the interaction between the positively charged chitosan molecules and the negatively charged bacterial cell membranes that causes alteration in the permeability, which results in the leakage of proteinaceous and other intracellular constituents [92]. The study revealed that the increase in ratio of chitosan (1:2 to 1:10) in the formulation, leads to greater diffusion of EDH across the membrane due to enhanced membrane permeation and thereby, the MIC of the formulation is lowered against *M. bovis* [93]. In short, this study clearly provided evidence that chitosan DPI formulation is a potential candidate for the delivery of anti-TB drugs with high permeability characteristic.

Another study conducted by Ahmad et al., in 2015 introduced a dimple-shaped chitosan carrier for EDH DPI to treat TB. A rough chitosan carrier surface can be used to improve the aerosol performance and reduce the Van der Waals forces of attraction between the drug and the carrier system [94]. In this study, the formulations were developed with constant drug ratio (2.25 mg) but varied in chitosan ratio. The surface areas of the chitosan increased proportionally from 11.0 to 15.4 m^2^/g with increasing chitosan concentration (0.1–0.4%). However, chitosan with 0.5 and 1% started to reduce in the surface area. This could be due to the large chitosan carrier size when the percentage of chitosan increases. Moreover, the dimples on chitosan surface are dependent on the inlet temperature, the concentration of the chitosan solution and the feeding rate during spray drying. The study revealed that the particles showed shrinkage on the surface when higher inlet temperature (150 °C) was used. In contrast, spherical shape particles were obtained when lower temperature (80 °C) was used. The obtained dimple-shape particles may due to the evaporation of water at high inlet temperature [95]. The results showed chitosan has no difference in molecular weight change (3.2 kDa) after spray-drying process to obtain dimple-shaped carrier particles. Chitosan carriers with an angle of repose (α) below 30° are said to be having good flowability [96]. In the study, all the chitosan with different percentages of concentration were below 30°. Based on the differential scanning calorimetry results, the EDH-loaded dimple-shaped chitosan DPI formulations were melted at 203 °C and became amorphous in nature. The amorphous material showed improved in solubility and faster dissolution rate that could lead to higher bioavailability of the drug [97]. Furthermore, the developed formulations were found to have uniform content with high DL at 99–107% except for formulation with EDH to chitosan carrier in 1:10 ratio did not exhibit uniform content with only 61% of loaded drug which could be due to the strong binding of drug to the chitosan carrier. The detachment forces drug from carrier partciles were increased when the drug-carrier ratio increased. The MMAD and FPF of the developed formulations showed the same results as discussed in the previous study done by Ahmad et al. in 2014, which exhibited good aerodynamic properties. The researchers concluded that mixing EDH with chitosan carriers in DPI formulation is a promising approach with better solubility and flowability for controlling TB [98].

### 5.4. Delivery Approaches of Rifamycins

Rifamycin is a transcriptional inhibitor known to have anti-TB activity. It is derived from the natural product of *Amycolatopsis mediterranei.* Rifamycin binds specifically to the β-subunit of RNA polymerase, which results in inhibition of the RNA synthesis in the bacteria. Rifampicin (RIF), rifapentine (RFP) and rifabutin (RFB) are the currently approved agents derived from rifamycins used for TB treatment [99].

#### 5.4.1. Rifampicin Formulation Approaches

In 2020, Khadka et al. formulated polymorphic forms of RIF-loaded dry powder formulation for pulmonary delivery. The spray-drying method and crystallization method were used to produce inhalable RIF formulations, where an amorphous formulation of RIF was produced by the spray drying method while a crystalline dihydrate and a pentahydrate formulation were developed by the crystallization method using different solvent systems. In the study, crystallization produced comparable higher process yield with smaller and fine particles (between 1.1–1.2 µm) compared to the spray-drying method. The amorphous formulation showed the least bulk and tapped density with 0.18 g/mL and 0.24 g/mL respectively, which expected to exhibit the best aerosol performance among all the prepared formulations. Solubility is one of the important parameters for inhalable dry powders. In the study, the amorphous formulation is shown to have higher solubility compared to pentahydrate formulations of RIF, which may due to the higher free energy state of the amorphous formulation. The in vitro deposition of all developed formulations was compared and the results showed that amorphous formulation had higher retention powders in the inhaler device and lower ED at 81%. However, amorphous formulation was shown to be highly deposited at the lower airway once the particles were emitted. Furthermore, the amorphous formulation showed lower MMAD with 2.3 µm than the crystalline formulations. This represented that amorphous formulation of RIF had better in vitro aerosolization capacity. The aerosolization capacity for both amorphous and crystalline dihydrate was found to be maintained after three months of storage at room temperature with low humidity. However, amorphous and crystalline pentahydrate were more prone to degradation and a decrease in drug content whereas the crystalline dihydrate formulation appeared to be the most aerosolization stable and showed the least degradation [100]. In short, the researchers concluded that RIF powders developed by crystallization and spray drying methods are promising candidates for further in vivo and in vitro studies.

Mehanna et al. formulated spray-dried RIF-carbohydrate nanocomposite (SD-RIF-NC) by using spray drying RIF nanosuspension with combination of carbohydrates (mannitol, maltodextrin and leucine) as the matrix formers. SD-RIF-NC made up with mannitol, maltodextrin and leucin at ratio 1:2:1 showed higher percentage of powder yield at 86.67%, in which the addition of mannitol and maltodextrin helps to protect particles from shrinkage and collapse. The addition of matrix former was also shown to minimize the fusion of inter-nanoparticle as the formulations with mannitol or maltodextrin resulted in the particle mean diameter reduced to 5.12 and 6.25 µm respectively, which are lower than the RIF nanosuspension (NS) without matrix former with 22.9 µm. Furthermore, the addition of leucine in the formulations showed a significantly lower particle diameter, with 3.47 µm and 4.35 µm in mannitol and maltodextrin formulations, respectively. This may due to the fact leucine tends to accumulate at the air-liquid interface due to its hydrophobicity, thus reducing the size of the droplets [101]. Additionally, the presence of leucine in the formulations was found to enhance the flowability of the powders as leucine has the ability to minimize powder cohesion, increase the inter-particle distance and total surface area, thus, reducing the inter-particular interactions [102]. The study demonstrated that addition of leucine in the formulation with mannitol showed 32.63°, 18.08% and 1.22 in term of angle of repose, Carr’s index and Hausner ratio respectively, whereas the maltodextrin formulation showed 37.58°, 24.23% and 1.32, which indicates both formulations exhibited the desired flow properties. Higher surface area (3.997 m^2^/g) with corrugated surface was observed in the formulations with added leucine. The addition of mannitol to the formulation with maltodextrin and leucine in ratio 1:2:1 exhibited rapid dissolution rate of RIF after 10 min with 100% of drug dissolved due to the better wettability of mannitol. The developed SD-RIF-NC showed good aerodynamic characteristics with 95.22% of EF, 65.41% of respirable particle fraction (RF) and 77.93% of effective inhalation index (EI) which are high for particles to deposit deep in the lungs [103]. Therefore, this study provided evidence for the use of RIF loaded carbohydrate spray-dried nanocomposite in DPI against TB with improved drug inhalation performance.

RIF-loaded mannosylated SLN assemblies (SLNas) were proven to be effective in targeting macrophages for TB therapy where methyl α-D-mannopyranoside (MP) was used due to the overexpression of mannose receptors on the infected AMs, thus this helps increase the selectivity of RIF-loaded SLNas towards the infected AMs [104]. Different lipid components were prepared by using palmitic acid (PA set) or tripalmitin (TP set), mixed with cholesteryl myristate and RIF to formulate RIF-loaded SLNas in the presence of sodium taurocholate (ST) as the surfactant and MP as the functionalizing agent. The developed SLNas showed a negative Z-potential (range from −44.40 to −63.7 mV) demonstrating the desired properties where the internalization process by AMs was enhanced. The incorporation of RIF into SLNas showed controlled released properties, in which only 30–50% of the drug was released within 3 h as compared to free RIF, in which nearly 90% of the drug was released within 1 h. The drug release profile demonstrated in SLNas should allow the quick uptake of drug by the AMs. RIF/PA with ratio 0.3:1 (PA1) and RIF/TP with ratio 0.5:1 and addition of ethanol as co-solvent (TP2E) showed the highest %EE and DL. Thus, both formulations were chosen to compare with the respective formulations without functionalization. RIF-loaded SLNas doses were found to be lower than the oral doses as the oral RIF caused an 80% reduction in cell viability with 100 µg/mL in 24 h, whereas PA1 and PA1 without functionalization at a dose of 0.25 mg/mL showed no significant toxicity on the J774 cell line. In addition, mannosylated SLNas provided better flowability, with a 31.18° angle of repose with reproducible ED. However, mannosylation could make the powder become cohesive due to moisture adsorption on mannose residues and high adhesion forces among mannose hydroxyl groups [105]. Therefore, the results of this study confirmed that RIF-loaded mannosylated SLNas are potential therapeutic nanocarriers requiring lower doses for TB treatment, in which the impaired respirability caused by powder cohesiveness will need to be investigated further.

Another advanced delivery system has been introduced to overcome the limitations of polymeric and lipid-based nanocarriers, nanolipomers. These nanolipomers possess the advantages of both polymeric and lipid-based nanocarriers, where the drug release could be controlled by the polymeric backbone and the loading and permeation of therapeutics within the carrier could be enhanced by the lipid component [106]. Absed on this concept, Mulla et al. developed RIF-loaded nanolipomer composites in the DPI platform formulated to increase the drug concentration at the targeted tissue and prolong the duration of drugs in the lungs which are the primary site of TB infection. Mulla et al., reported that RIF-loaded nanolipomer composites could be prepared via microemulsion-spray drying by using PA as lipid component and polycaprolactone (PCL) as polymeric component. The results showed that the particle size was increased from 382.5 nm to 561.8 nm, where 2:1 *w/w* ratio of PA: PLC showed the highest in particle size with %EE at 73.14% which was the highest as well. This indicates that as the concentration of lipid component (PA) increases, a larger particle size is formed, and hence, the greater the EE. However, the particle size of lipomer composites required for pulmonary delivery should below 100 nm to obtain better therapeutic outcomes, therefore, further research is needed to reduce the particle size of nanolipomer composites to the optimum size. The solid-state properties revealed that the nanolipomer composites were in an amorphous state, in which the drug expulsion from the composites will be minimized. The RIF released from the nanolipomer composites in simulated lung fluid showed rapid drug dissolution by an initial burst release of RIF in the first 24 h followed by sustained release of RIF over 96 h. The initial burst release profile would be favorable to achieve an early therapeutic plasma concentration of RIF [107]. Hence, the results suggested that development of RIF-loaded nanolipomer composite in DPI platform could increase the residence time of drugs in the targeted tissues.

Another study conducted by Singh et al., introduced a novel phospholipid lipospheres to deliver RIF as an alternative to conventional therapy. Phospholipids (PLs) was proven effective to be used in pulmonary delivery as they are biocompatible and biodegradable with improve particle migration to the lung for local and systemic action [108]. The formulated RIF-loaded lipospheres showed good flow properties as the angle of repose, Carr’s index and Hausner’s ratio fall within the acceptable range at 33.5°, 14% and 1.11, respectively. The MMAD and GSD of lipospheres were 2.72 and 3.28, respectively, with 77.61 FPF and 80% ED. The in vitro aerosol performance results revealed that lipospheres were deposited in the lower regions of the airways. This implies that a combination of low MMAD and high FPF indicates that phospholipid liposphere can be delivered deep in the lung regions with high drug deposition. Additionally, RIF-loaded lipospheres showed controlled release properties with drug residence up to 36 h and the ability to escape from mucociliary lung clearance when tested in vivo. In the in vivo studies, it also revealed that the RIF concentration from nebulized lipospheres was lesser in the non-targeted tissues than from the pure RIF suspension [109]. In general, it can be concluded that the use of phospholipid lipospheres in DPI demonstrates superiority in targeting and delivering RIF to the deep lung over the conventional treatment.

Earlier, we had reported the delivery of RIF-loaded into the vesicular structure of liposomes [110,111]. This alternative approach of delivering RIF into the respiratory tract was achieved through delivering liposomal suspensions, where the liposomes were prepared following chloroform film method using cholesterol and soybean l-infinity-phosphatidylcholine. Formulated unilamellar and multilamellar liposomal vesicles (200–300 nm) were further freeze-dried and can be aerosolized for the purpose of application. The formulation was reported to possess considerable stability compared to the drug solution and liposomal suspension [110]. Further analysis of these RIF-loaded liposomes with 50% EE were found to have 3.4 µm size, possessing non-toxic characteristics to different respiratory cells. The formulation was found to produce no stimulation of inflammatory cytokines, where these liposomal delivery was found to possess a suitable carrier to inhibit growth of *M. bovis,* with MIC of 0.2 µM compared to 0.8 µM of free RIF. This increased efficacy could be explained by the increased uptake and prevent the intracellular growth of *M. bovis,* because of negatively charged vesicles [111].

Besides, RIF loaded-PLGA nanoparticles were developed with the incorporation of arginine and leucine as diluents to improve the RIF content in primary nanoparticles, in a study carried out by Takeuchi et al. In this study, RIF-loaded PLGA nanoparticles showed higher EE at 65.2% when the volume and pH of the aqueous phase were 40 mL and pH 4, respectively. This can be attributed to the fact the solubility of RIF is reduced at low pH. Thus, reducing the volume and pH of the aqueous phase improved the RIF content in the PLGA nanoparticles. The nanoparticles produced in this study were within the range of 190.4 nm to 207.0 nm and showed no significant change in the mean volume diameter. In the in vitro release studies, nanoparticles prepared with low aqueous phase volume (40 mL) and low pH (pH4) showed suppression of the initial burst release. Furthermore, the study showed that with the addition of arginine and leucine, the developed nanocomposite particles inside were non-spherical particles with a shape similar to large porous particles. The presence of arginine and leucine at the ratio of 1:20 in RIF loaded PLGA nanoparticles produced higher FPF at 32.63% with approximately 200 nm diameter as the nanoparticles produced without diluents. This indicates that nanocomposite particles prepared using diluents are suitable for deep inhalation in the lungs. The results from this study showed that inhalable nanocomposite particles developed using arginine and leucine were useful to deliver RIF for pulmonary delivery, however, these nanocomposite particles will need to further investigation to prove their safety to treat TB [112].

In the mentioned study by Takeuchi et al. the addition of l-leucine to RIF-loaded PLGA spray dried microparticles has shown to be showed suitable for use in pulmonary delivery with deep lung deposition [113]. Takeuchi and co=workers carried out another study to examine the effect of RIF loaded PLGA microparticles on the FPF and the phagocytotic ratio of AMs with the presence of l-leucine. In this study, RIF-loaded PLGA microspheres and microparticles added with different concentrations of leucine were compared, showing diameters within the range from 5.9 µm to 11.7 µm, where RIF-loaded PLGA microsphere had the smallest diameter (5.9 µm). Microparticles showed about 5–6 µm in average particle size which indicates their suitability to be used for inhalation. The FPF (<4.7 µm) increased by 4.3 to 6.9 times and decreased one-third in tap density when leucine was added to the aqueous phase. The maximum FPF (43.7%) was observed in microparticles with 0.2% of leucine in the aqueous phase. However, there is no significant difference between the geometrical particle diameter and density among the microparticles formulated with leucine concentrations from 0.1 to 0.3%. It was found that the shape of the particles had a great effect on the FPF. The study reported that non-spherical microparticles with 0.2% leucine have higher phagocytotic ratios on NR8383 cells at 66.8% compared to spherical particles. Moreover, the concentration of RIF obtained in AM was 0.34 µg/mL which meets the MIC for TB treatment [113]. Therefore, non-spherical PLGA dried spray microparticles could effectively deliver RIF to the AMs with the presence of l-leucine.

Functionalized nanocarriers with peptides for pulmonary delivery play an important role in transporting high drug payloads to the targeted site specifically [114,115,116]. Tuftsin is one of the interesting peptides that is constituted with threonine (T), lysine (K), proline (P) and arginine (R) [117]. It has been reported that tuftsin peptide can selectively recognize the specific receptors that are available on the surfaces of macrophages and monocytes, and thus enhance the uptake by the cells [118]. Consequently, Carneiro et al., formulated functionalized RIF-loaded nanostructured lipid carriers with tuftsin-modified peptide (NP-pRIF) to improving the therapeutic effects. The particle size of RIF-loaded nanostructured lipid carriers without functionalized (NP-RIF) increased from 210 nm to 285 nm with the presence of tuftsin-modified peptide. However, the low PDI value (0.18) of NP-pRIF assured a monodisperse nanosystem. The addition of peptide to NP-RIF showed no interference with the incorporation of RIF as the percentages of EE and DL still remained high at 81% and 7.4 mg/100 mg, respectively. In this study, asymmetrical flow field-flow fractionation (AF4) showed more reliable and accurate results compared to dynamic light scattering (DLS), in which fractionation in AF4 technique allows particle sizes to be reported based on their proportionality whereas the average particle sizes may be influenced and overestimated in DLS as it is performed on the whole sample. Based on the AF4 results, 50% of the NP-pRIF particles were 255 nm in size which were smaller than the average DLS particle size at 293 nm. Therefore, the results revealed that both NP-pRIF and NP-RIF present a narrow and homogeneous particle size distribution. Both NP-pRIF and NP-RIF also showed high stability under low temperature, where no significant change was observed in particle sizes, PDI and zeta potential when the formulations were kept at 4 °C for 2 months. In addition, both NP-RIF and NP-pRIF released around 22% and 18% of the drug, respectively, in 72 h in vitro which indicates that drug release from nanostructured lipid carriers was controlled. NP-pRIF was more effectively taken up by macrophages with high payload of drug compared to NP-RIF. The mean fluorescence intensity (MFI) results revealed that functionalized nanostructured lipid carrier has higher MFI (93.4) than nanostructured lipid carrier without functionalization (37.3), which is equivalent to a 2.5-fold increase in internalization. The concentration required to inhibit *M. tuberculosis* growth in vitro for pure RIF was 1.0 µg/mL whereas NP-RIF and NP-pRIF required only 0.48 µg/mL. These results providing that the attachment of tuftsin-modified peptide on NP-RIF improved antimycobacterial activity by enhancing the penetration of NP-pRIF through the cell wall [114]. Therefore, RIF-loaded nanostructured lipid carriers represent a promising approach for better TB management with low dosage requirements, consequently reducing the side effects.

In 2017, Rawal et al., formulated RIF-loaded chitosan DPI to enhance the therapeutic approach for alveolar TB. The formulation containing RIF, chitosan and TTP was prepared by a freeze-drying method. The drug and the excipients were compatible with each other. The particles of the formulations presented spherical shapes within a range of 124.1 ± 0.2 nm to 402.3 ± 2.8 nm. TPP concentrations were reported to have positive effects on the particle size of the formulation in which the higher the concentration of TPP, the larger the size of the formulation particles. Besides, the particle size distribution of the formulation with the PDI of 0.195 to 0.594, indicating a narrow range. The positive zeta potential demonstrated that the particles of the formulation had higher chances to be taken up by AMs as they can form electrostatic interactions with negatively charged sialic acid present on the surface of the AMs [119,120]. The study also revealed that the increase in acetic acid concentration will lead to an increase in EE, whereby the EE of the formulation was increased from 32.8 ± 1.7% to 72.0 ± 0.1% when the acetic acid concentration increased to 1% due to the complete solubility of chitosan. In addition, the best-optimized formulation possesses a MMAD of 3.3 ± 0.18 µm with the GSD of 2.0 ± 0.04, indicated that the formulation is suitable for deep lung delivery and the size distribution is narrow. The best-optimized formulation also had the FPF of 33.27 ± 0.87% which demonstrated good lung deposition and providing better-targeted site delivery to the lungs. For the in vivo studies carried out on male Wistar rats, a huge increase of 1.5-fold and 2.1-fold in lung drug concentration were seen in the rats treated with RIF-loaded chitosan-TPP formulation compared to conventional DPI (prepared by mixing micronized RIF with coarse and fine lactose) and orally administered RIF. Other than that, the increase in RIF-loaded chitosan-TPP formulation drug elimination half-life also suggested that this formulation possess sustained released properties of drug. In summary, the RIF-loaded chitosan-TPP formulation can significantly improve the aerosolization properties, and increase targeted site concentration, hence, increasing treatment efficacy [121].

Recently, Berkenfeld et al., have developed inhalable formulations of RIF by spray drying supersaturated aqueous solutions. The RIF aqueous formulations (RIF-Aq) and suspension formulation (RIF-SP) showed a highly collapsed, thin-walled microsphere whereas the RIF isopropyl alcoholic solution formulation (RIF-IPA) (reference formulation) presented as collapsed spheres with an irregular shape. All the formulations produced amorphous particles. For the aerodynamic performances, RIF-Aq and RIF-SP demonstrated an extremely high EF of >98% while RIF-IPA had a lower EF of 69.1 ± 2.3%. The MMAD of all formulations were range from 1.4–2.0 µm with the GSD value of 2.9–3.4. In addition, the FPF of the RIF-Aq and RIF-SP were 80–89% whereas RIF-IPA showed a lower FPF of approximately 37%. Although the FPF of RIF-IPA was relatively low compared to RIF-Aq and RIF-SP, however, all of these formulations possess the ability to deliver RIF into the lower respiratory region. For the stability testing, the storage at low temperature successfully minimized the degradation of the amorphous formulation. Generally, all the formulations possess the potential to improve the TB treatment efficacy by targeted site delivery and their long-term stability, cytotoxicity and treatment efficacy of the formulation should be further investigated [122].

#### 5.4.2. Rifabutin Formulation Approaches

In 2014, Pai et al. developed and evaluated chitosan microparticle-based DPI formulations for RFB prepared by using a spray drying method. The formulations were composed of RFB, chitosan, TPP and lactose. The microparticles were presented corrugated and wrinkled surfaces. The yield of the formulation ranged between 14.37–20.17%. Noteworthily, there was no significant difference in yield for RFB-loaded microparticles when different concentrations of TPP were used. The moisture content of the formulation was lower than 2%, which was acceptable as a higher moisture content led to the aggregation of particles and hence an increase the particle size and decrease in the aerodynamic performance. The particle size of RFB-loaded microparticles was within 1.146 ± 0.832 µm and 1.769 ± 0.588 µm and all formulations, regardless of the TPP:chitosan ratio, showed positive zeta potentials ranging from 18.1–23.2 mV. The larger particle size was obtained when the TPP concentration increased as the viscosity of the solution to be spray dried increased and resulted in the formation of larger droplet sizes during the spray drying process [123]. Other than that, the increase in particle size was also due to the increase in the amount of TPP incorporated into the same volume of droplets [123]. The PDI of 0.258–0.95 also indicated that the formulation had a unimodal distribution. The DL efficiency for RFB-loaded microparticles ranged from 70 to 89% whereby the amount of drug loaded into the microparticles decreased when TPP was increased due to the increased extent of cross-linking. It was also suggested that the increase in TPP will decrease the availability of chitosan binding sites for RFB as some of the binding sites interact with TPP ions, hence, leading to a reduction in DL efficiency. In swelling studies, a swelling index reduction with an increase in TPP concentration was observed. In the in vitro drug release studies, RFB-loaded microparticles showed more sustained release of drug compared to free RFB in which RFB-loaded microparticles released 90% of the drug by the 96 h mark. Furthermore, the MMAD and GSD of RFB-loaded microparticles were near to 5 µm and 1.2, respectively, with the FPF of >30%. These results demonstrated that RFB-loaded microparticles were suitable for deep lung delivery. Moreover, the internalization of RFB-loaded microparticles by AMs were observed in the in vitro AMs uptake study which the internalization increased with an increased in the number of RFB-loaded microparticles. In the in vivo toxicity tests carried out on female Sprague Dawley rats, no significant toxicity was observed to the lungs of the rats. Overall, the RFB-loaded microparticles can possibly provide benefits of lower dose and reduced frequency of treatment compared to oral administration, however, further studies are required on the efficacy of these microparticles against *M. tuberculosis* [124].

**Figure 5:**
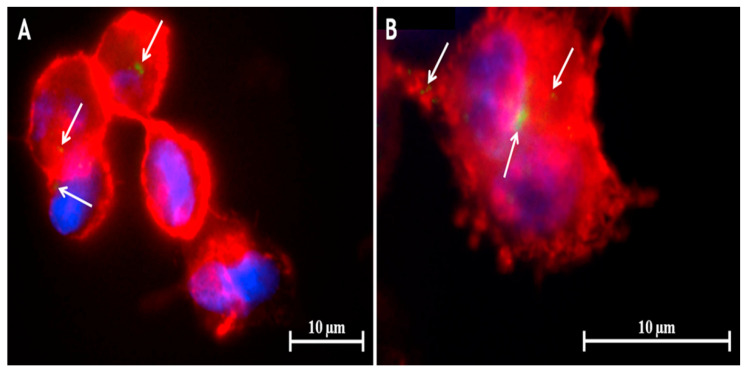
Fluorescence micrographs of (**A**) GDB-SLNs and (**B**) GTT-SLNs, which SLNs were labelled with green colour (coumarin-6) whereas actin and nuclei of macrophages were labelled with red (rhodamine phalloidin) and blue (DAPI dye), respectively. (Adopted from [127]).

Gaspar et al., formulated RFB-loaded SLNs dry powder formulations for pulmonary tuberculosis via lyophilisation. SLNs composites loaded with RFB were developed by using different lipids which were glyceryl dibehenate (GDB) and glyceryl tristearate (GTT). Tween 80 was used as a surfactant. The RFB-loaded SLNs presented a spherical shape and the loaded RFB was in the amorphous state. The particle sizes of RFB-loaded GDB-SLNs formulation and RFB-loaded GTT-SLNs formulation were 121 ± 2 nm and 299 ± 10 nm, respectively, with a PDI range between 0.17–0.20. Besides, the EE for the RFB was around 89.9% for GDB-SLNs and approximately 81.0% for GTT-SLNs. In the DLS thermal analysis, the results suggested that both of the SLNs formulations were able to resist the harsh formulation conditions as the particle size variations were within the nanosize range after applying the heat up to 90 °C [125,126]. In addition, propidium iodide, a dead cell indicator, were applied to the cells to monitor the membrane integrity and the results demonstrated that the cells treated with the RFB-SLNs formulations do not have disrupted plasma membranes. Moreover, in the intracellular SLNs uptake studies, both of the SLNs formulations were internalized by THP-1 cells efficiently. According to Figure 5, the SLNs labeled with coumarin-6 (green) were successfully taken up by THP-1 cells (rhodamine-phalloidin was used as a marker of actin (red) and DAPI dye was used to stain the nuclei (blue)). The results showed that the THP-1 cells had taken up 25.9 ± 8.6% of RFB-loaded GDB-SLNs and 6.3 ± 0.9% of RFB-loaded GTT-SLNs after 1 h and the uptake were increased to 46.3 ± 3.0% and 25.6 ± 9.3% after 24 h, respectively [127]. The uptake difference between the GDB-SLNs and GTT-SLNs was due the difference in particle size as the smaller size particles can be phagocytized more readily by macrophages [128]. Furthermore, This study demonstrated that >95% of RFB were released from the GDB-SLNs and GTT-SLNs after 24 h which a burst release of 65% of RFB was observed in the first 30 min, followed by a slower exponential release of the remaining drugs. This study proved that lipid nanoparticles are potential carrier system for direct lung delivery of antituberculosis agents [127].

#### 5.4.3. Rifapentine Formulation Approaches

In 2016, Parumasivam et al., formulated RFP-loaded PLGA microparticles for TB inhalation therapy by a spray drying method or oil-in-water (O/W) single emulsion solvent evaporation. The D_50_ for all formulations ranged between 1.3–1.5 µm. The spray-dried particles presented smooth, spherical shapes whereas O/W single emulsion evaporation formed mixtures of smooth and corrugated, spherical particles. This study suggested that neither the ratio of lactide to glycolide nor the differences in molecular weight could explain the morphology of the particles, but the powder production method showed certain effects on the morphology. Furthermore, there was a huge difference in the DL efficiency between the different production methods. The DL efficiency was close to 100% with the spray drying method while <10% was found in O/W emulsion evaporation method. The low DL in O/W emulsion particles was due to the partition of RFP from the dispersed oil phase into the external water phase [129]. In the dynamic vapor sorption analysis, the results demonstrated that a lower mass change was observed in the formulation with higher lactide proportion compared to the formulation with lower lactide proportion, however, all the formulations were proven stable after exposure to humidity. For the aerosolization properties, the ED for all formulations was found to be >80%. Besides, the FPF of the spray-dried formulations were slightly higher than the O/W emulsion formulations, which were in a range of 52–57% and 40–45%, respectively. The lower FPF of O/W emulsion formulation may be attributed to the high cohesive force between the particles and the turbulence inside the inhaler device was unable to deagglomerate the powders effectively [130]. Moreover, the MMAD of all formulations ranged between 2.3 and 3.1 µm, with a span value of around 2–3 which indicated that the formulations were mildly polydisperse and able to reach deep into the lungs. The in vitro dissolution studies revealed that all the formulations showed a biphasic drug release profile in which an initial burst release through diffusion and dissolution mechanism was observed from the surface of the microspheres, followed by a slow sustained release of RFP via diffusion. In addition, the cellular uptake profile demonstrated that all formulations regardless of lactide:glycolide ratio and molecular weight could be uptaken by THP-1 cells efficiently. The results also revealed that the formulations with high lactide:glycolide ratio can be internalized by macrophages more efficiently compared to low lactide:glycolide ratio formulations due to the greater hydrophobicity which enhanced the tendency of particles to adsorb to the macrophages’ surface [131]. In cytotoxicity studies, none of the PLGA formulations exhibited cytotoxicity to the THP-1 and H549 cell lines, therefore, PLGA-RFP formulation is another potential option for the treatment of pulmonary TB [132].

Patil-Gadhe et al., developed a proliposomes DPI formulation containg RFP by using a spray-drying method. The SEM revealed that the pure RFP was present as needle-shaped crystals whereas spray-dried RFP and RFP-proliposomes formulations possess spherical particles with rough and smooth surfaces, respectively. The average mass median diameter for RFP-loaded proliposomes was around 7.73 µm, with a range of 0.89–1.3. Furthermore, the angle of repose between 23 and 31° and low tapped density of between 0.105 and 0.677 g/cc also indicated that the RFP-loaded proliposomes formulations had good flow properties which may improve their aerodynamic performances. The EE of the proliposomes formulations were from 22.71% to 72.08%, while the RFP:HSPC ratio was found to have certain impact on improving the EE. The increase in RFP: HSPC ratio will increase the amount of lipid available for RFP encapsulation which further increases the hydrophobicity, resulting in enhanced encapsulation. The liposome vesicle size was found to be in a range of 442 ± 4.6 nm and 803 ± 10.6 nm, which indicated that the proliposome formulations can be taken up by macrophages efficiently. The vesicles within 200–600 nm can be taken up by macrophages for intracellular killing whereas the larger vesicles are responsible for extracellular killing [133,134]. As for the aerodynamic properties, no significant difference in MMAD of all proliposome formulations was observed within a range of 1.56–5.26 µm. Besides, the FPF (<4.6 µm) of the proliposome formulations fell between 60–92%. These aerodynamic properties indicated that the proliposome formulations were suitable for lower respiratory tract drug delivery. For the in vitro release study, the proliposome formulations released around 90% of RFP at the end of 24 h whereas pure RFP and spray dried RFP were released completely in 8 h and 4 h, respectively. This emphasized that the RFP proliposoms formulation possesses drug sustained-release properties. In addition, the in vivo studies carried out on albino rats demonstrated that the RFP concentration in lungs was found to be more than 2-fold higher in all the spray-dried formulations compared to pure RFP. The results also revealed that the mean residence time and elimination half-life of the RFP increased by 7-fold in prolipsome formulations compared to spray dried RFP. Overall, this study showed that spray-dried proliposomes DPI formulation have potential in enhancing tuberculosis treatment efficacy, however, further investigation on the cytotoxicity towards the respiratory associated cells and long-term stability of the formulation are required [135].

### 5.5. Delivery Approaches of Formulations Containing Therapeutic Combinations

The combination of different anti-TB agents in anti-TB treatment is one of the big challenges to improve patients’ compliance due to the fact multiple drugs and frequent dosing is required. Thus, novel approaches for delivering multiple anti-TB drugs concurrently have great potential in improving treatment efficacy and patients’ compliance.

Rodrigues et al., developed a dual antibiotherapy of TB mediated by inhalable locust bean gum (LBG) microparticles via a spray drying method. The formulation comprised two first-line anti-TB drugs, INH and RFB, together with LBG. Product yields in a range of 60–70% were obtained, with loading capacities of 8.2% and 4.4% for INH and RFB, respectively, which are near the theoretical maxima (INH:8.70% and RFB:4.35%). The LBG-based microparticles were found to be irregular in shape with convoluted surfaces. However, the morphology of the LBG microparticles was not associated with the loaded drug. In addition, the Feret’s diameter of INH-RFB-LBG microparticles was 1.15 ± 0.51 µm. Besides, INH and RFB obtained the MMAD equal to 6.2 µm and 5.8 µm which were larger than 5 µm due to a certain amount of powder deposited on the high stages on the impactor. It had been suggested that the high MMAD was due to the insufficient deaggregation of INH-RFB-LBG microparticles. The FPF of the formulation was found to be 38%, which pointed out that a proportion of LBG-based microparticles have an aerodynamic diameter <5 µm. A high ED of 92% was obtained, stipulating the good flowing capacity of the LBG-based microparticles. In the in vitro release study, both of the anti-TB drugs were released rapidly from the LBG microparticles as a result of the high solubility of both the drug and polymer in aqueous media. However, a slow release of drugs in vivo was predicted due to the reduced amount of fluid in the alveoli. Most importantly, the AMs uptake study showed that LBG-based microparticles have a very high macrophage uptake efficiency of 95–100% in human monocytes (THP-1 cells) and NR8383. The high uptake efficiency in NR8383 resulted from the mannose and galactose units in LBG which favoured the recognition by the C-type lectin receptor on macrophages surface [136,137,138]. Hence, LBG-based microparticles need to be optimised in the future in the aspect of MMAD, and particle morphology to obtain better aerosolization performance and treatment efficacy [139].

In one of our studies on human volunteers, we have reported that the administration of dry powder forms of anti-tubercular agents is safe. In this context, we prepared dry powder delivery systems of INH, RIF, PZA and levofloxacin, where the in vitro tests revealed them to have uniform size for lung delivery and also uniformity of delivered dose. During the evaluation of these formulations in human volunteers, they were found to be safe as there was an absence of pro-inflammatory cytokines in sputum or any other adverse events [140].

Tse et al., had developed matrix embedded formulations for combination spray-dried particles composing two other first-line anti-TB drugs which were PZA and RIF. The formulation was developed by using highly branched cyclic dextrin (HBCD) through a spray-drying method. After that spray drying process, the particles showed porous, spherical appearance. The yield of the drug-loaded HBCD particles was 70.91 ± 6.19%, which was considered acceptable. Moreover, the MMAD of the particle of drug-loaded HBCD obtained were range between 2.28 ± 0.01 µm and 2.89 ± 0.13 µm which were suitable for deep lung deposition. The FPF of the PZA and RIF in the HBCD formulations with different ethanol concentration were in a range of 30.0 ± 2.8%–48.7 ± 6.9% and 31.0 ± 5.6%–51.2 ± 6.5%, respectively. However, the results suggested that the concentration of ethanol did not have a significant effect on the MMAD and FPF of the formulations. The ED of the formulations achieved a high value of around 90%. It had been suggested that the small, porous particles were attributable to the high FPF and ED of the formulations. For the drug dissolution profile, the dissolution rate of both PZA and RIF in the PZA/RIF/HBCD formulation showed a huge improvement compared to the untreated PZA and RIF. The enhancement in the dissolution profile was due to the formation of amorphous drugs after the spray drying process. Therefore, HBCD formulation is a potential and highly feasible candidate for the novel delivery of anti-TB drugs [141].

**Figure 6:**
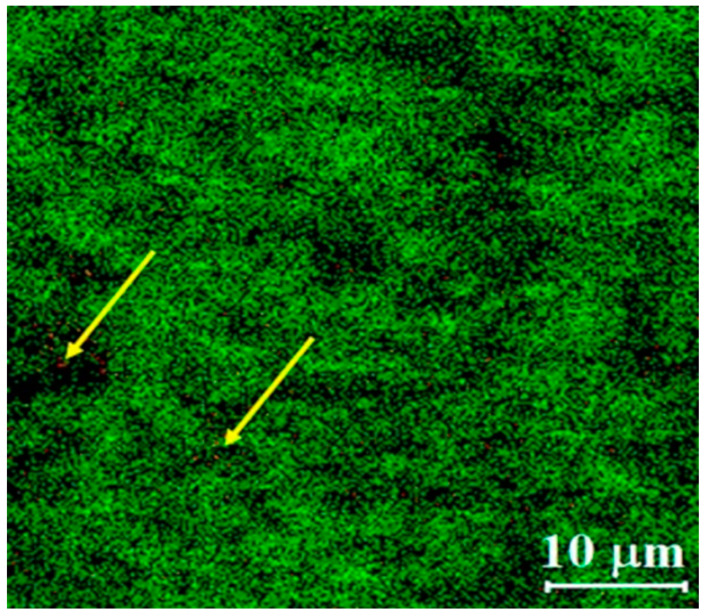
The distribution of KNM and RIF on the particle surface. The green spots represent RIF whereas KNM was represented by red dots. (Adopted from [145]).

Momin et al., investigated the effect of co-spray drying hygroscopic kanamycin (KNM) with hydrophobic RIF to improve the aerosolization of the kanamycin powder for treating respiratory infections. The SEM images revealed that the particles of the combination formulation appeared wrinkled and flake-shaped, whereas the particles of spray-dried KNM presented smooth and spherical shapes. The morphology of the combination formulation was thought to be able to reduce inter-particulate contact, hence enhancing the aerosolization properties. The KNM-RIF combined formulation afforded a high yield of 75.9% which was much higher than the 30.3% yield of spray-dried KNM alone. The high yield of the combined formulation was due to the presence of hydrophobic RIF on the surface of the particles which reduces the wall deposition during the spray drying process [142]. Noteworthily, the surface of the particles in the combined formulation was enriched with hydrophobic RIF, as shown in Figure 6, which may also be a cause of the improvement in aerosolization performance of the powders by reducing particle interactions [143,144]. Besides, the average content of KNM and RIF in the combined formulation was ranged between 95.8–104.8% which falls within the pharmacopoeial range of 75–125% for mass balance for preparations of inhalation formulations. The particle size distribution for spray-dried KNM, RIF and combination of KNM-RIF showed a narrow distribution in which the particle volume diameter (D_50_) for all types for spray-dried formulation was lowered or equal to 3.84 µm with a span from 1.07–1.57. Although the D_50_ for spray-dried KNM was significantly larger than the combined formulation, it was suggested that the practical difference between each spray-dried formulations is unlikely to be significant as all of the formulations showed a particle size of <5 µm which highlighted the ability for particles to reach into the deep lungs. All the spray-dried formulation had high ED values larger than 83.9%. Furthermore, the FPF of the combined formulation was >77.6% compared to the FPF of spray-dried KNM alone which was <29.5%. The MMAD of the combined formulation was 1.4–1.9 µm and the GSD was range from 2.1–2.9, which again indicated that the combined formulation had a narrow particle size distribution and was suitable for lower respiratory deposition. The tapped density of the combined formulation was found to be significantly lower than the spray-dried KNM, which were 0.274 ± 0.03 g/mL and 0.655 ± 0.04 g/mL, respectively. For the cytotoxic studies, the formulations were shown to be nontoxic to both Calu-3 and A549 cells as the cell viabilities after treated with the formulations were not significantly different compared to before treatment with the formulations. Overall, the surface enrichment of KNM by hydrophobic RIF showed improvements in the aerosolization performance [145].

Rossi et al., successfully loaded RIF, INH and verapamil (VPM) into low molecular weight sodium hyaluronate nanocomposite respirable microparticles to manage antibiotic resistance in the treatment of mycobacterial pulmonary infections. The yield of the product was 50.1% and the adherence of the powders to the cyclone and collector was observed. The hydrophilic nature of HA that likely entraps water in its structure increased the water content of the powder which in turn increased the adhesive force and led to the adherence of the powders to the cyclone and collector [146]. Even though the moisture content of the powder was high, however, the powder was found to be stable at room temperature. The particles produced were present in irregular shapes with slightly rough surfaces. Besides, the EE of the INH, RIF and VPM was 48.75 ± 4.32%, 77.52 ± 0.11% and 80.45 ± 4.80%, respectively. The low EE of INH was due to its hydrophilic nature, whereby INH tends to remain in the aqueous phase during the precipitation of polymeric nanoparticles. The D_50_ of the of the microparticles was 0.94 ± 0.15 µm with a span value of 1.79 ± 0.17 which suggested that the particle size distribution of the formulation was narrow. The aerodynamic performance of the powder was investigated with Turbospin^®^ and RS01^®^ in which the ED was 74.25 ± 8.20% and 50.40 ± 15.83% of the loaded dose, respectively. Furthermore, a very high FPF for all drugs was obtained in both devices (>76% for Turbospin^®^ and >90% for RS01^®^). This demonstrated that the sodium hyaluronate nanocomposite microparticles were suitable for inhalation and able to deposit in the alveolar region. In dissolution studies, INH was found to release completely within 24 h whereas both RIF and VPM had a slower release rate, which was only 60% release for RIF and 70% for VPM after 40 h. The slower release rate of RIP and VPM was due to their low solubility in water which is 2.5 mg/mL and 83 mg/mL, respectively [147,148]. Moreover, both of the formulations (with or without VPM) showed antimycobacterial activity with a combined MIC_99_ of 0.25 µg/mL and a combined MIC of 32 µg/mL for the susceptible strain H37Rv and two-drugs resistant strains, respectively. In an ex vivo study, both formulations were able to reduce bacterial viability to less than 20% irrespective of the drug resistance profile at day 3 post-infection [149]. This formulation thus showed strong scientific evidence of suitability in enhancing the macrophage elimination of mycobacteria, irrespective of the drug-resistance profile.

Recently, Momin et al., formulated a DPI formulation combining bedaquiline (BDQ) with PZA for latent and drug-resistant tuberculosis by using a spray-drying method. The formulation (BPL) was composed of BDQ, PZA and 20% (*w/w*) of l-leucine. The yield of the BPL was 57.2%, which was the highest compared to spray-dried BDQ and PZA alone. The particles of BPL were porous, with a spherical appearance and a size of 1.7 ± 1.0 µm [150]. The formation of porous particles had been suggested to be due to the presence of highly surface-active l-leucine [151]. Besides, the geometric diameter also indicated that the BPL formulation was suitable for deep lung delivery. The drug recovery for all the spray-dried formulation was >82% which is within the acceptable range of 75 to 125% [152]. Other than that, the ED of the formulation was found to be higher than 80%. Moreover, the FPF of BPL was 68.5% and 66.9% for BDQ and PZA alone, respectively, which is significantly higher than the spray-dried BDQ (31.3%) and PZA (5.1%). The low FPF of spray-dried BDQ and PZA alone was due to both morphology and aerodynamic diameter of the particles. The MMAD of BPL was ranged between 2.9–3.2 µm whereas both spray-dried BDQ and PZA alone possess the MMAD of 5.9 ± 0.1 µm and 6.0 ± 0.1 µm, respectively. It had been suggested that the porous particles with an aerodynamic diameter of 1–5 µm are highly aerosolisable and able to deliver into the lower respiratory region and can be uptaken by AMs easily [153,154]. Furthermore, the GSD of all the formulations were range from 1.9–2.7, indicated that the particle size distribution was narrow [155]. The BPL powder was found to be stable as the aerosolization properties remained unchanged after one-month of storage under high humidity conditions. Overall, BPL possesses good aerosolization properties, thereby, is a potential pulmonary delivery candidate for treating latent and drug-resistant tuberculosis [150].

The same year, Rangnekar et al., developed a similar formulation with the addition of moxifloxacin (MX). In general, the addition of MX showed no significant difference compared to BPL, however, a slight increase in the aerosolization properties (FPF and MMAD) was observed [156].

### 5.6. Delivery Approaches for Vaccines

The Bacille Calmette-Guerin (BCG) vaccine is an approved vaccine for the prevention of TB infections. BCG was derived from a live attenuated strain of *M. bovis* and still remains the most widely used vaccine globally [157]. Based on a report by Thakur et al., the current TB vaccine candidates in the clinical pipeline, including BCG vaccine, are in suspension form and administered through injection. Liquid vaccine dosage forms have the disadvantages of instability during storage and requiring refrigeration during manufacturing and distribution [158]. Moreover, BCG provides highly variable protective effects against pulmonary TB among the human population (children, adults and the elderly). Revaccination with BCG in populations vaccinated with BCG at birth showed no increase in protection against TB infection [159]. As a result, the development of new efficacious and safer vaccines is needed to prevent and control the spread of TB disease.

Thakur et al. developed a dry powder-based H56/CAF01 vaccine with dimethyldiocta-decylammonium (DDA) bromide and the glycolipid trehalose-6,6′-dibehenate (TDB) by using a spray-drying method. The multistage subunit vaccine antigen, H56, is a fusion protein with early protective *M. tuberculosis* antigens ESAT-6 and Ag85B, and the latency-associated protein Rv2660c. The cationic adjuvant formulation 01, CAF01 activates dendritic cells, resulting in a long-livedvaccine that induces T-helper type 1 (Th1) and Th17 responses, and shows favorable protective efficacy when combined with H56 in numerous preclinical studies. The results of the study indicated that co-spray-dried, reconstituted H56/CAF01 formulations show a slight decrease in particle size with z-average in the range of 225 nm to 685 nm whereas H56/CAF01 in liquid formulations ws in the range of 370 nm to 770 nm. The reconstituted H56/CAF01 formulations after spray drying also showed identical cell-mediated and humoral immune responses as the spray-dried H56/CAF01 formulations, showing no significant difference in the production of antigen-specific interferon-γ (IFN- γ) as well as the ability to induce polyfunctional Th1 and Th17 responses when compared to non-spray-dried formulations at 5 µg of H56. Additionally, increasing H56 to 20 µg in both spray-dried and non-spray-dried formulations showed no increase in IFN-γ production. This finding could be due to lower T-cell receptors affinity threshold that was found to be regulating CD4+T cell differentiation after vaccination. Thus, the researchers concluded that co-spray-dried H56/CAF01 is a thermostable, safe and effective vaccine for TB [158].

The application of biomaterial components in either tissue engineering or vaccine delivery system is known to have adjuvant effects, as measured by the observed enhancement of the host immune response [160,161,162]. Encapsulation of BCG and *M indicus* pranii (MIP) in bio-polymeric alginate microparticles as dry powder aerosol (DPA) were found to provide better protection against *M. tuberculosis* in a study conducted by Nagpal et al. [163]. The developed formulations were found to be stable at temperatures up to 40 °C after 6 months of storage. High secretion of interleukin-12 (IL-12) was observed in MIP encapsulated in alginate particles (MEAP) (6851 ± 11 pg) and BCG encapsulated in alginate particles (BEAP) (3721 ± 20 pg) immunized cells, indicating the activation of dendritic cells was comparable higher than those immunized with liquid aerosol MIP and BCG. Similarly, the release of TNF-alpha, interferon gamma (IFN gamma) and the proliferation of allogenic T cells were significantly higher when treated with MEAP and BEAP. The MEAP and BEAP were efficiently engulfed by bone marrow derived dendritic cells (BMDCs) with maximum uptake after 24 h and co-localized with lysosomes. The results also revealed that MEAP/BEAP-activated BMDCs showed higher up-regulation of CCR7 receptor compared to MIP/BCG activated BMDCs. It has been reported that up-regulation of CCR7 results in effective presentation of antigen to the T cells [164]. The in vivo immune response studies reported that MEAP and BEAP produced relatively high proliferation and IFN-gamma in splenocytes and mediastinal lymph node cells. Additionally, the average body weight of the mice immunized with MEAP and BEAP after 130 days of treatment was significantly higher compared to MIP and BCG immunized mice. However, no mortality was observed in any of the immunized groups. Alginate coated MIP and BCG DPA provides better protection in mice when infected with *M. tuberculosis* H37Rv than liquid aerosol as the bacterial load in the lung and spleen were lower in MEAP and BEAP immunized groups. From the histopathological result, mice immunized with BEAP/MEAP showed minimal granulomatous lesions whereas mice immunized with aerosol BCG/MIP showed relatively diffuse infiltrate of granuloma and severe necrotic with unorganized granulomatous lesions was observed in the unimmunized mice. These findings demonstrated that BEAP/MEAP immunized animals have minimal lung pathology as compared to BCG/MIP animals, respectively. In short, alginate-coated BCG/MIP formulated in DPA are very promising vaccine candidates for TB with better protection against *M. tuberculosis* H37Rv [163].

In addition, the developed inhalable dry powder of AERAS-402 reported by Jin et al. by using mannitol-based powder (mannitol-cyclodextrin-trehalose-dextran, MCTD) demonstrated a more stabilized formulation for TB treatment. AERAS-402, which is one of the adenovirus-vectored 35 (Ad35) TB vaccines, had the ability to induce an immunity response with high levels of antigen- specific polyfunctional CD4 T cells and CD8+ T cells in humans [165]. MCTD stabilizer showed better thermostability as a higher glass transition temperature value (*Tg*) (97.09 °C) was obtained in the thermodynamic behavior study whereas the corresponding values for trehalose and mannitol with polyvinyl pyrrolidone (PVP) were 50.55 °C and 85.78 °C, respectively. Spherical spray dried powders produced from mannitol-based formulation had a narrow size distribution (3.2–3.5 µm) and exhibited good aerodynamic properties. The mannitol formulation showed the least water content (1.28%) among all the prepared formulations. However, inhalation of dry powder mannitol alone was reported to increase the mucociliary clearance in the lungs, and thus MCTD formulation was chosen for development of dry powder vaccines. Moreover, the MCTD formulation demonstrated higher resistance to water adsorption, with only a 1.80% *w/w* increase in water content whereas trehalose formulation showed a 8.70% *w/w* increase in water content when exposed to high humidity (70%) conditions. The MCTD formulations showed no decrease in the percentage of inhalable particles (IP < 5 µm) after 28 days of storage at 37 °C. All formulations suffered more than 1.5 (maximum 4.9) log loss of the viral infectivity whereas the MCTD formulation showed only 0.83 log loss after spray drying. The process yield for mannitol-based formulations were more than 30%, in which leucine formulation showed the least, with only 4.3%. The inhalable MCTD dry powder AERAS-402 showed good physical stability, with only 0.12 log loss of virus activity when stored at 37 °C for 5 weeks. Therefore, the researchers concluded that MCTD is a leading candidate for the development of dry powder formulations of AERAS-402 TB vaccine with better stability profile.

In another study carried out by Tyne et al., the researchers formulated inhalable spray-dried TLR2-targeted powder vaccines, in which lipokel was used to deliver antigens to the immune cells for induction of Th1 responses. Additional protective effect was seen in immunizing with a fusion of cutinase-like proteins (Culps) 1 and 6, reported by Shanahan et al. [166]. Therefore, Culps1-6 fusion and MPT83 were used as *M. tuberculosis* antigens, conjugated with lipokel by spray dying with mannitol in this study. Mice immunized with either lipokel only or Culps1-6-Lipokel showed significantly influx of neutrophils, myeloid DCs, monocytes and pulmonary macrophage in the lungs, and the responses still remained high in Culp1-6-Lipokel group by day 7, which indicates the sustained increase of protein-lipokel vaccine in the frequency of activated DCs and macrophages. The developed MPT83-lipokel conjugate also showed sustained DCs response. Moreover, both CD4+ and CD8+ effector T-lymphocytes were significantly increased in the mediastinal lymph node (MLN) after vaccination with the respective Culps1-6-lipokel and MPT83-lipokel. The induction of antigen-specific, IFN-gamma producing T-lymphocytes was enhanced as well as the antigen-specific IgG responses were stimulated after immunized with the protein-lipokel conjugate. Both Culps1-6-lipokel and MPT83-lipokel conjugated powder vaccines provided significant protection against *M. tuberculosis*-infected mice when compared to unimmunized mice. Thus, the results of this study confirmed that TLR2 targeted lipokel conjugated to recombinant *M. tuberculosis* antigens are potential novel pulmonary dry powder vaccines for the prevention of TB [166].

The various pulmonary delivery strategies against tuberculosis have been summarized in Table 1. From the available reports, it can be inferred that pulmonary application of anti–tubercular drugs using advanced drug delivery system can enhance the treatment efficacy with improved safety profiles.

## 6. Beneficial Aspects of Proliposomal Dry Powder Inhaler over Other Nanocarriers in Treatment of Tuberculosis

Among all the novel drug delivery systems, liposomes are the most effective, researched and broad approach which possess the ability to enhance therapeutic efficacy and reduce the adverse effects of the encapsulated drugs. However, the physical and chemical instability of liposomes limits the shelf life, hence, restraining the therapeutic applications [168]. Therefore, proliposomes which were designed by Payne et al., in 1986 are used to overcome the problems related to conventional liposomes aqueous suspensions, especially the instability caused by aggregation, fusion and phospholipid hydrolysis which affects the shelf life of the formulations [169]. Proliposomes are dry, free-flowing particles which will disperse to form a multi-lamellar, isotonic liposomal suspension when in contact with water or biological fluids [169,170,171]. Therefore, they have the potential to be developed into inhalable dry powder inhalers for delivering antimicrobial drugs [172]. 

In the treatment of pulmonary tuberculosis, proliposomal dry powder inhaler formulations provide targeted drug delivery into the lungs, with large exposure followed by rapid onset of action which increases the antitubercular drug concentration in the lungs, thereby reducing the plasma concentration. For instance, the RFP concentration in lungs was found to be two times higher in proliposomes formulation than pure RFP [173]. These properties provide the advantages of enhancing treatment efficacy, and at the same time, improved patient compliance as the drug-associated systemic toxicities are reduced [73,93,133,173]. In addition, proliposomes DPI formulation of INH showed greater anti-mycobacterial activity against *M. bovis* compared to free INH as INH-proliposomes can be efficiently internalized by infected AMs [50]. Rojanarat et al. reported similar in 2012 results for a levofloxacin-proliposomes formulation against *M. tuberculosis* [133]. Thus, a lower dose of anti-tubercular drug might be used in proliposomal formulations to treat pulmonary tuberculosis which results in reduced treatment-related toxicity [50,133]. Furthermore, the proliposomal DPI formulation possesses controlled, sustained release properties [168,170,173,174]. A study carried out by Patil-Gadhe et al., revealed that RFP-proliposomes formulation showed prolonged release of RFP for over 24 h compared to pure RFP, which was released completely in 8 h. The mean residence time and the elimination half-live also showed significant increases (around 7-fold) in the RFP proliposomes formulation compared to pure RFP [173]. The sustained drug release properties of proliposomes formulation provide the benefits of maintaining targeted site drug concentration above MIC for a longer period which can reduce the dose and administration frequency, thereby, reduce dose-related adverse effects, increase tolerability and minimize the progression of resistant strains [175]. Similar to liposomes, proliposome formulations hve a high EE towards lipophilic, hydrophilic, amphiphilic and charged hydrophilic anti-tuberculosis drugs due to the unique structure of the liposomes vesicles [176]. Lipophilic drugs can be entrapped into phospholipid bilayer whereas hydrophilic drugs will be incorporated into the aqueous phase [177,178]. After the entrapment of drugs in proliposomes, the solubility of the hydrophobic drug increases and this leads to an increase in drug bioavailability which enhances the treatment efficacy. Moreover, the encapsulated drugs are protected from direct contact with external environments either during storage or delivery process, thus, minimize the degradation of the drugs and increase the effectiveness of the anti-TB therapy [176]. Overall, proliposomes are a better alternative drug carrier to the liposomal vesicular system with higher stability which makes it easier to handle and manufacture. Therefore, increased the cost-effectiveness of the anti-TB therapy.

## 7. Safety Concern of Dry Powder Inhaler Formulation

Other than treatment efficacy, safety is also considered as one of the most important criteria in the development of novel drug delivery systems. In vitro or in vivo cytotoxicity studies are essential to evaluate the safety of inhalable dry powder formulation and provide valuable information for the formulation development. The potential cytotoxicity of the dry powder formulations can be determined by using lung-derived cells such as alveolar macrophages or lung epithelial cells. Moreover, several animal models have been used in in vivo studies to investigate the safety of dry powder inhaler formulations such as mice, rats and guinea pigs.

### 7.1. Safety of Lipid-Based Carriers Intended to Lung Delivery

The lipid-based carriers such as proliposomes, liposomes, SLN, and NLC, showed enhancement in safety profile with reduced cytotoxicity in both in vitro and in vivo studies. For example, Rojanarat et al. tested PZA-proliposome formulations with 90% and 20% porous mannitol on human bronchial cells (Calu-3), human lung adenocarcinoma cells (A549) and alveolar macrophages (AMs) (NR8383). The cell viability of Calu-3 cells was more than 80% after tested with 50 µg/mL of PZA-proliposome formulation containing 90% of porous mannitol which suggested that the formulation was nontoxic to the cells. Both PZA-proliposome formulations at concentration 25–500 µg/mL showed no toxicity on A549 cells and NR8383 cells as the cell viability of both cell lines was more than 80%. Moreover, the results showed that PZA-proliposomes formulations did not activate AMs to release inflammatory mediators as the amount of interleukin-1β (IL-1β) and tumor necrosis factor-α (TNF-α) were less than 5 pg/mL and 10 pg/mL, respectively, which fall outside of the detectable dose range (12.5–400 pg/mL). Nitric oxide (NO) produced by NR8383 cells was less than 0.2 µM [73]. Similar results were shown in another study carried out by the same group, where LEV-proliposome formulations with 90% and 20% of porous mannitol were also tested on Calu-3 cells, A549 cells and NR8383 cells. The results showed that both proliposome formulations at various concentration (LEV content at 0.25–5 µg/mL) showed no toxicity to respiratory system-associated cells as the cell viability of the tested cell lines were more than 80% in vitro. The NR8383 cells were not activated by both LEV-proliposome formulations as the amount of IL-1β and TNF-α produced were less than 10 pg/mL. Both LEV-proliposome formulations also produced less than 0.2 µM of NO [133]. The negligible amounts of inflammatory mediators and NO produced in response to PZA-proliposome and LEV-proliposome formulations were insufficient to show secondary inflammatory [133]. Furthermore, the cell cytotoxicity studies carried out on AMs after exposure to INH-proliposomes formulations with 90% and 20% mannitol content developed by Rojanarat et al. in 2011 showed no significant toxicity for a concentration of less than 2.5 mg/mL. AMs maintained nearly 100% of cell viability after the exposure to less than 2.5 mg/mL of INH-proliposomes formulation and started to reduce when the concentration increased to more than 2.5 mg/mL. It has been suggested that the concentration of 2.5 mg/mL (toxic concentration) is unlikely to occur in the respiratory tract via pulmonary administration and is 5000× higher than the MIC of INH against tuberculosis bacillus. For normal human bronchial cells (NHBE) and small airway epithelial cells (SAEC) which are more sensitive to INH-induced toxicity, the cell viabilities of 100% were obtained when exposed to INH-proliposomes formulations with 90% mannitol content. However, the cell viabilities were reduced to around 70% in all the concentrations for INH-proliposomes formulations with 20% mannitol content. These results demonstrated that mannitol was not toxic to the respiratory cell lines but possesses a stabilizing effect, which can affect the cell viability. Lastly, similar to LEV-proliposomes and PZA-proliposomes formulations, INH-proliposomes formulation did not activate AMs to produce IL-1, TNF- and NO at a level that would lead to secondary inflammation [50]. Apart from that, ethambutol-loaded SLNs developed by Elham et al. showed no toxicity on A549 cell line in the in vitro cytotoxicity studies, which indicates this formulation is safe to further test its efficacy in vivo [89]. Besides, Gaspar et al. found that RFB-loaded SLNs formulations showed similar results on A549 and Calu-3 cells as the cells’ viabilities remained >80% after exposure to the SLNs formulations for 24 h [127]. In addition, the cytotoxicity studies of both NP-RIF and NP-pRIF formulations that were formulated by Carneiro and team demonstrated no toxicity on J774 A.1 cells as the cell viability was more than 70% after 24 h of incubation [114]. On the other hand, Maretti et al. developed RIF-loaded SLNas and investigated for their safety and dosage. The results indicated that the SLNas doses lower than the oral doses as the oral RIF caused 80% of reduction in cell viability with 100 µg/mL in 24 h, whereas pulmonary SLNs at the dose 0.25 mg/mL showed no significant toxicity on J774 cell line. Mannosylated SLNas was found to be suitable for alveolar macrophage passive targeting and drug maintained in nanocarriers before macrophage internalization. However, phagocytosis and respirability impaired by powder cohesiveness indicates the need of active targeting [105].

### 7.2. Safety Concern of Polymeric-Based Nano/Micro Carriers for the Treatment of Tuberculosis

Regarding the safety profile of polymeric-based nano/micro carriers, Ahmad et al. carried out cytotoxicity tests on the chitosan of EDH DPI formulations. The results showed that the chitosan formulation exhibited low toxicity on A549, Calu-3 and NR8383 cell lines, with viability values between 80 and 90%, while EDH DPI without chitosan carrier was shown to have slight toxicity on lung cell lines, which indicated that the presence of chitosan may reduce the toxicity of the DPI formulations [93]. Another example carried out by Rawal et al. highlighted the RIF-loaded chitosan-TPP formulation was safe to use in anti-TB delivery system in comparison to free RIF. This could be explained by the fact that the cell viability of J774 cells was significantly decreased after the exposure of free RIF at the concentration >0.125 mg/mL for 6–12 h, whereas the cell viability of 80–90% was obtained after the exposure of RIF-loaded chitosan-TPP formulation at all concentrations (0.125 mg/mL, 0.25 mg/mL and 0.5 mg/mL) for 6–12 h. Moreover, for the in vivo studies carried out on male Wistar rats, both RIF-loaded chitosan-TPP and conventional DPI formulation showed no toxicity to the lungs of the tested animals while the orally administered RIF solution showed severe toxicity [121]. Similarly, chitosan microparticles-loaded with INH were evaluated for safety. The cytotoxicity tests on alveolar murine (AMJ2-C11) and J774.1 macrophages cell lines demonstrated that low molecular weight chitosan microparticles did not show cytotoxic activity on the macrophage cell linage. In summary, low molecular weight chitosan microparticles are promising carriers for INH pulmonary administration [79]. A similar result was shown in RFB-loaded chitosan microparticles developed by Pai et al., which no significant toxicity was observed on the lungs of the Sprague Dawley rats [124]. Besides, another polymeric DPI formulation, namely RFP-loaded PLGA microparticles formulated by Parumasivam et al., exhibited no cytotoxicity to the THP-1 and H549 cell lines [132]. Last but not the least, Tse et al. have provided further evidence on the safe use of polymeric DPI formulation, in which the matrix embedded formulation comprising PZA and RIF using HBCD possesses no observable cytotoxic effect against A549 cells as the cell viability remained unchanged after being treated with the formulation [141].

In terms of other micro/nano systems, Mehanna et al. have found that the incorporation of RIF in the nanocomposite showed low cytotoxicity on the A549 cells when the concentration of RIF used is less than 1 mg/mL. The RIF concentration reported to be effective in TB therapy was 5.35 µg/mL, which indicates that nanocomposites were safe to use for delivering the drug [103]. Furthermore, the results of a cytotoxicity study of combined RIF, INH and VPM-loaded low molecular weight sodium hyaluronate nanocomposite respirable microparticles carried out by Rossi et al. on human monocyte-derived macrophages (HMDM) showed no toxicity for up to 5 days as the viability of the macrophages remained at 100% after 5 days and started to decrease at day 7. In another study Momin et al. reported that both PZA and BDQ in BPL formulations showed no toxicity to A549 cells for up to 100 µg/mL. Besides, in Calu-3 cells, PZA again showed no toxicity for up to 100 µg/mL, however, BDQ was found to be toxic even at 75 µg/mL. Interestingly, for BPL, both of the A549 and Calu-3 cells able to maintain cell viability of higher than 80% up to 100 µg/mL. This demonstrated that the combined formulation has the ability to reduce BDQ toxicity [150]. Similar results were shown in the cytotoxicity evaluation of the same formulation with the addition of MX, which was out by Rangnekar et al. It was suggested that the reduction of BDQ cytotoxicity might be due to the lower proportion of BDQ in BPML when compared to spray-dried BDQ alone [156]. Lastly, contrary to other DPI formulations discussed above, the combined INH and RFB-loaded LBG-based microparticles showed an effect in decreasing A549 cell viability to approximately 60% [139]. Thus, further optimization in the aspect of safety profile are required for LBG-based formulation.

In another study the applicability of rifapentine crystalline DPI was evaluated for safety and results indicated that it is well tolerated by macrophages and pulmonary tissue up to the dose of 50 μM [167]. Similarly, Momin et al., investigated the effect of co-spray drying hygroscopic KNM with hydrophobic RIF for lung infection and its safety. The cytotoxic study showed that the formulations is nontoxic to both Calu-3 and A549 cells as the cell viabilities. Cell viability after treated with the formulations were not significantly different compared to before treated with the formulations. Overall, the surface enrichment of KNM by hydrophobic RIF showed improvements in aerosolization performances [145].

In summary, it could be said that DPI formulations are relatively safe to use via the inhalation route and can be considered potential candidates for alternative tuberculosis treatment, which can enhance treatment efficacy.

## 8. Clinical Aspect of Dry Powder Inhaler Formulation against Tuberculosis

The optimistic advantages of anti-tubercular DPI formulation via pulmonary administration have been proven with the increasing number of studies in the research field. Some of the anti-tubercular DPI formulations are undergoing clinical trials to evaluate their pharmacokinetic profile, efficacy, tolerability and safety. Capreomycin is the first anti-tuberculosis agent available in microparticle DPI formulation. This formulation was undergoing a phase 1, single-dose, dose-escalating clinical study on 20 healthy adult volunteers using a handheld inhaler. Overall, the formulation was well-tolerated, and no serious adverse effects were observed. However, mild to moderate transient coughing was noticed in 20% of the subjects. A serum drug concentration above 2 µg/mL which is the MIC for *M. tuberculosis* was reported after a single dose of 300 mg was administered. The results suggested that inhaled therapy has the potential to be the MDR-TB treatment regimen [179]. Other than that, PureIMS, a clinical-stage biopharmaceutical company in July 2020 has initiated phase 1 clinical trial of Amikacin via Cyclops™ (an amikacin DPI formulation) to evaluate the pharmacokinetic properties and local tolerability of the formulation in patients with drug-susceptible TB. Previous studies have shown that amikacin is well-tolerated via the pulmonary route [180,181].

## 9. Expert Opinion on Drug Delivery on Tuberculosis Treatment

DPIs have become very popular as an attractive platform for drug delivery, especially for respiratory diseases such as TB. In this paper, we have reviewed a variety of DPI formulations which incorporate either a single drug or a combination of multiple anti-TB agents to effectively manage the rising tide of TB in the world. Good aerosolization properties is one of the most important criteria for inhalable DPI formulations to understand and estimate the in vivo deposition manner of powders after being inhaled. Therefore, most of the DPI formulations are developed with the addition of a carrier to minimize the dose variability and increase the dosing accuracy by improving the flowability of the drug particles. Besides, the handling of powder during manufacturing operation can be improved and the deposition of the drug into the lungs can be controlled. In addition, nano/micro technologies often incorporated into DPI formulations to further enhance the solubility of the drug, hence, increase bioavailability and treatment efficacy. In the existing literature search, there are numerous excipients such as l-leucine, mannitol, chitosan, lactose etc, had been investigated as carrier to be used in DPI formulations.

Leucine has been widely used as carrier in DPI formulations as it provides advantages in improving the aerosolization performance of anti-TB drugs. Leucine is more likely to accumulate at the air-water interface and inhibits the penetration of solvent vapor during the spray drying process. This property of leucine affects the morphology of the particles produced, which results in a reduction of particle density. Leucine also increases the process yield as it has the ability to reduce the adhesion forces between the particles and the cyclone wall during spray drying processes. Based on the literature reviews, mannitol and lactose are other commonly used sugar-based carriers for DPI formulations. Lactose possesses biocompatibility and biodegradability characteristics which increased its useability in DPI formulation, whereas, the good wettability of mannitol increases the dissolution rate of the incorporated drugs. Both mannitol and lactose are beneficial in increasing the flowability of the formulations. However, porous mannitol is a more suitable carrier for DPI formulations compared to mannitol. It is because mannitol tends to form smooth spherical particles, which will lead to the agglomeration of the particles and resulted in FPF reduction. Furthermore, the phospholipid-based carriers such as proliposomes can be developed with the addition of soybean phosphatidylcholine and cholesterol to mannitol/porous mannitol. The phospholipid bilayer in the phospholipid-based carrier is biocompatible with human body components. This characteristic enhanced the bioavailability of the hydrophobic drugs, which in turn enhanced the treatment efficacy. In addition, enhanced anti-mycobacterial activity with lower drug toxicity could be achieved by incorporating anti-TB drugs into phospholipid-based carrier as the drug can be delivered to the targeted tissues and internalized by AMs efficiently. Furthermore, polymeric carriers such as chitosan, PVA and PLGA are also suitable to be used in DPI formulations due to their sustain drug release characteristics. Based on the literature, the drug-released profile from a polymeric carrier showed an initial burst release, followed by a sustained drug released pattern. The initial burst released of drug able to boost the drug plasma concentration to the desired level in a short duration, whereas the sustained released properties maintain the plasma concentration for a longer period. The drug released profile has the potential in reducing drug dosing and frequency which subsequently reduces the treatment-related toxicity. Most importantly, the literature had proven that these carriers are safe to be used when incorporated in DPI formulations.

## 10. Conclusions

In conclusion, the treatment of TB has become complicated with the emergence of drug-resistant TB. Pulmonary delivery of anti-TB agents with dry powder inhaler formulations seems promising for the treatment of or vaccination against TB and has been developed to overcome the limitations of conventional treatments such as the high dose required, frequent dosing and systemic adverse effects. This review summarized the use of dry powder inhaler (DPI) formulations which are more stable than solutions or suspensions for the delivery of either drug only or synergistic drug combination to treat TB in the deep lung with higher doses. The advantages of dry powder inhaler formulations for therapeutic intervention in treating TB is clearly evident, as shown in the results of many studies, such as improvement in formulation stability, better uptake by the alveolar macrophages (AMs), possesses sustain release profile and reduced side effects. However, further study in the expectation of long-term safety profile and efficacy of the DPI formulation need to be carried out to ensure patients’ safety and better treatment outcome.

## Figures and Tables

**Table 1:** Pulmonary drug delivery strategies in the treatment of tuberculosis.

Objective	Route of Administration	Type of Formulation	Dosage Form	Method of Preparation	Excipient Used	Cell Line/Animal Model	Outcome	Source
To investigate the effect of adding l-leucine and using an ethanolic solvent on the physicochemical properties and aerodynamic behaviours of nano spray-dried PZA-l-leucine powders.	Pulmonary	Dry powder inhaler	Nanocarriers	Spray drying method	l-leucine Valine	NA	The co-spraying of PZA with an optimum l-leucine content using the nano spray drying technique increased the FPF by around 2-fold and reduced MMAD and GSD values. EF of formulations ranged from 86.5–98.4%. Drug loading of pure PZA and PZA-leucine formulations were in the acceptable range of 83.1–99.7%. Optimised formulation has the FPF of 33.0 ± 4.1% and the MMAD of 2.83 ± 0.04 µm.	[66]
To optimize a formulation of PZA as large porous particles for pulmonary delivery by adjusting spray drying parameters	Pulmonary	Dry powder inhaler	Microcarriers	Spray drying method	DPPC Hyaluronic acid Ammonium bicarbonate D,l-leucine	Male Sprague Dawley rats	The formulation EF and a spray-dried yield were 99 ± 3% & 47.3 ± 0.8%, respectively. The drug content was 34.9 ± 2.2%. MMAD of the PZA-LPPs was 4.1 ± 0.2 µm with FPF of 40.1 ± 1.0%. The AF and GSD were 29.6 ± 3.1% and 2.16 ± 0.16 respectively. PZA-LPPs via intratracheal insufflation increased the PZA concentration in ELF by 1.28-fold compared to intravenous administration. The ratio of ELF concentrations over plasma concentrations was 2-fold greater after intratracheal administration than after intravenous administration.	[67]
To develop and optimize phospholipid-based PZA spray-dried inhaler powders.	Pulmonary	Dry powder inhaler	Phospholipid-based microparticles	Spray drying method	DPPC DSPE-PEG2k l-leucine	NA	The ED for formulations was >70%. FPF of phospholipid-based PZA formulation was 73.2 ± 4.0%. Formulation possess MMAD of 2.5 µm and GSD values of 1.75 ± 0.1 The phospholipid-based PZA spray-dried inhaler powders increased the FPF by approximate 8.6-fold.	[72]
To develop PZA-proliposomes in dry powder aerosol form for delivering drugs to AMs.	Pulmonary	Dry powder inhaler	Proliposomes	Spray drying method	Porous mannitol	Calu-3 cell lines A549 cell lines NR8383 cell lines	The co-spray drying of PZA with optimum fraction of porous mannitol obtained an inhalable dry powder with MMAD of 4.26–4.39 µm and FPF of 20–30%. ED of the formulations were larger than 80% with the EE of 44.6 ± 0.5%. PZA-proliposomes formulation shown to be less toxic towards respiratory cell line and did not activate AMs to release inflammatory mediators.	[73]
To develop proliposomes powder containing INH in a dry powder aerosol form	Pulmonary	Dry powder inhaler	Proliposomes	Spray drying Method	Mannitol	NHBE cell lines SAEC cell lines AMs	Proliposomes mean vesicle size range of 300–400 nm with MMAD less than 5 micrometer. INH-proliposomes possess better antimycobacterial activity against *M. bovis*-infected AMs than free INH.	[50]
To prepare and characterize INH-loaded chitosan microspheres for pulmonary delivery	Pulmonary	Dry powder inhaler	Polymeric microparticles	Spray drying Method	Chitosan TPP l-leucine Lactose	NA	The size of microspheres and EE was found in the range of 3.6 to 5.2 µm and 87.5 to 72.1% respectively. ED (%), FPF (%) and MMAD of the formulation was 88.34 ± 2.16; 63.2 ± 3.2; and 2.79 ± 0.22 respectively. INH-loaded chitosan microspheres possess sustained release properties and is suitable for deep lung delivery.	[76]
To prepare and characterize spray dried inhalable powders containing chitosan nanoparticles for pulmonary delivery of INH.	Pulmonary	Dry powder inhaler	Polymeric nanoparticles	Spray drying Method	Chitosan TPP l-leucine Lactose	NA	The FPF for the formulations with leucine is significantly higher than the formulations without leucin, however, low FPF found in the formulation with maltodextrin. INH-loaded chitosan nanoparticles possess sustained release properties. Leucine exhibited significant effects on physical and aerosolization properties of spray dried powders.	[78]
To develop INH-loaded microparticles with 50–190 kDa chitosan as promising nontoxic carriers for pulmonary delivery.	Pulmonary	Dry powder inhaler	Polymeric microparticles	Spray drying Method	Chitosan TPP	J774.1cell lines AMJ2-C11 cell lines	Microparticles was in the range of 3.2–3.8 micrometer and showed positive zeta potentials between 17.7 mV and 29.8 mV. Microparticle entrappment was more than 89%. The concentration of INH in mucosa treated with INH-loaded chitosan microparticles was reported to be 6-fold higher than the mucosa treated with free INH. The low molecular weight chitosan microparticles did not show cytotoxic activity to alveolar cell linage.	[79]
To develop pulmonary delivery of antitubercular drugs using spray-dried lipid-polymer hybrid nanoparticles.	Pulmonary	Dry powder inhaler	Lipid-polymer hybrid nanoparticles	Spray drying Method	Soy Lecithin DSPE-PEG2k PLGA Mannitol	J774A.1 cell lines Male mice	The particle size of INH-LPNs was 111.81 ± 1.2 nm with a polydispersity index (PDI) of 0.189 ± 1.4. The MMAD of 2.49 ± 0.12 µm and FPF of 64.1 ± 1.2% showed good aerosolization properties. ED of the INH-LPNs was 88.34 ± 1.42%. INH-LPNs possess sustained, controlled drug release properties. INH-LPNs have higher uptake efficiency by AMs compared to free INH.	[84]
To assess pulmonary DPI using EMB-loaded SLNs for TB treatment	Pulmonary	Dry powder inhaler	Solid lipid nanoparticles	Homogenization and ultrasonication	Compritol Tween 80 Poloxamer 407	A549 cell lines	EE of formulation was 99.04%, PDI was 0.253, drug loading (DL) was 29.71% and particle sizes were below 60nm. Carr’s index and Hausner ratio of EMB-loaded SLN were 6.43 and 1.068 respectively, showed good flow property. EMB-loaded SLNs showed biocompatibility and good stability without toxicity observed. EMB-loaded SLNs with mannitol formulation demonstrated better flowability with spherical shape and less adhesion compared to mannitol-free formulation. EMB-loaded SLNs possessed controlled released profile which can be advantageous for reduced the dose frequency and minimizing drug side effects.	[89]
To investigate the acceptance of EDH containing chitosan in the form of dry powder formulation for further in vivo studies to target AMs for the treatment of TB	Pulmonary	Dry powder inhaler	Polymeric nanoparticles	Nanospray drying method	Chitosan	Calu-3 cell lines NR8383 cell lines A549 cell lines	EDH containing chitosan DPI formulations showed deep deposition in the lungs with 2 µm MMAD. Cytotoxicity of EDH DPI formulations is reduced with the present of chitosan. EDH DPI formulations with higher ratio of chitosan exhibited lower MIC values of <1 μg/mL with higher antimicrobial activity.	[93]
A dimple-shaped chitosan carrier was developed to deliver EDH in the form of DPI to the infected lungs	Pulmonary	Dry powder inhaler	Polymeric microcarriers with nanosize drug	Spray drying method	Chitosan	NA	EDH-loaded dimple-shaped chitosan showed improved in aerosol performance as the MMAD (2.3–2.9 µm) and FPF (34–42%) results were fall within the acceptable range with ED more than 80%. EDH-loaded dimple-shaped chitosan showed better solubility with amorphous in nature.	[98]
To investigate the polymorphic forms of RIF for inhaled high dose delivery in TB treatment	Pulmonary	Dry powder inhaler	NA	Spray drying method and crystallization method	NA	NA	Crystalline pentahydrate and dihydrate obtained higher powder yield (ranged from 79.2 to 83.3%) compared to amorphous formulation of RIF (74.8%). Amorphous formulation is expected to exhibit the best aerosol performance with the least bulk and tapped density at 0.18 g/mL and 0.24 g/mL respectively. Amorphous formulation results in better in vitro aerosolization capacity with lower MMAD (2.3 μm) and particles are highly deposited at the lower region of the airway with emitted dose of 81%. Crystalline dihydrate formulation of RIF showed the least of oxidative degradation among all the prepared formulations upon storage for 3 months.	[100]
To formulate RIF loaded carbohydrate spray dried nanocomposite in DPI for TB	Pulmonary	Dry powder inhaler	Microcarriers with nanosize drug	Antisolvent precipitation-ultrasonication method, spray drying method	Carbohydrate matrix formers (mannitol, maltodextrin and leucin)	A549 cell lines	RIF loaded carbohydrate spray-dried nanocomposite improved in inhalation performance evidence by high values of %EF (95.22%), %RF (65.41%), and %EI (77.93%).	[103]
							Nanocomposite-based RIF loaded dry inhalable powder showed high drug loading of 89.3–99.2%, a favorable particle size of 3.47–6.80 μm and uniform size distribution. IC_50_ of RIF nanocomposite was significantly higher than that of free RIF which was safe on lung tissue.	
To develop RIF loaded mannosylated solid lipid nanoparticle for the active targeting of macrophage in TB therapy	Pulmonary	Dry powder inhaler	Solid lipid nanoparticles	Melt emulsifying technique, freeze drying method	Cholesteryl myristate Palmitic acid/ tripalmitin	J774 cell lines	Mannosylated SLNas showed improved cell internalization in which the functionalized formulation was recognized by the mannose receptors located on the infected AM and thus quickly phagocyted by AM. The mean concentration of RIF by inhalation route was 113 times higher in the AM than oral RIF. Mannosylated SLNas provided better flowability with 31.18 º angle of repose with reproducible emitted dose.	[105]
To develop respirable RIF-loaded nano-lipomer composites by microemulsion-spray drying for pulmonary delivery	Pulmonary	Dry powder inhaler	Nanolipomers	Microemulsion-spray drying	Palmitic acid (PA) Polycaprolactone (PCL)	NA	The particle sizes of the lipomer ranged between 382.5 ± 6.033 to 561.8 ± 4.965 nm with a narrow polydispersity index (0.315 ± 0.023 to 0.424 ± 0.033) and zeta potential (−32.5 ± 1.206 to −26.5 ± 1.211 mV). Rifampicin entrapment efficiency was between 61.25 ± 1.049 to 73.14 ± 1.048%. Nanolipomer composite with higher content of PA showed greater in entrapment efficiency. The developed RIF-loaded nano-lipomer composites revealed its amorphous state.	[107]
							RIF-loaded nanolipomer composite showed initial burst release of RIF followed by controlled release profile.	
To formulate RIF loaded phospholipid lipospheres carrier for pulmonary application	Pulmonary	Dry powder inhaler	Phospholipid-based lipospheres	Spray drying method	Lipoid S-75	Sprague Dawley rats	RIF-loaded phospholipid lipospheres exhibited desired flow properties. Low MMAD (2.72), high FPF (77.61) and high ED (80%) that observed in phospholipid lipospheres demonstrated deep lung deposition of the formulation. RIF-loaded phospholipid lipospheres has 5-fold higher in drug residence time in lung compared to RIF suspension. RIF concentration from phospholipid lipospheres in non-targeted tissues was lesser from pure RIF suspension.	[109]
To improve RIF content in primary nanoparticles and to investigate arginine and leucine for the preparation of nanocomposite particles with low hygroscopicity	Pulmonary	Dry powder inhaler	Polymeric nanoparticles	Emulsion solvent evaporation method	PLGA	NA	The RIF content in nanoparticles was found to be 1.36-fold higher in the aqueous phase volume of 40 mL with pH4. RIF-loaded PLGA nanoparticles showed suppression in initial burst release at low volume and pH of aqueous phase. The addition of arginine and leucine as diluents in the ratio of 1:20 produced higher FPF at 32.63%. The diameter of RIF-loaded PLGA nanocomposites particle found to be nearly 200 nm.	[112]
To study the effect of leucine on the FPF and phagocytotic ratio of AM of RIF loaded PLGA microparticles	Pulmonary	Dry powder inhaler	Polymeric microparticles	Spray drying method, O/W emulsion, lyophilization	PLGA	NR8383 cell lines	RIF loaded PLGA with addition of arginine to leucine ratio of 1:20 showed the highest FPF (32.63%) with nanoparticles diameter around 200 nm. RIF-loaded PLGA nanoparticles exhibited higher EE at 65.2% in pH 4 of 40 mL aqueous phase. Microparticles showed about 5–6 µm in the average particle size. FPF (<4.7 µm) increased by 4.3 to 6.9 times and one-third decreased in tap density with leucine.	[113]
To synthesis RIF NLC functionalized with tuftsin-modified peptide to improve TB treatment	Pulmonary	Dry powder inhaler	Nanostructured lipid carriers	Microemulsion technique	Oleic acid Stearic acid Tween 80 Phospholipon 80H	J774 A.1 cell lines	Particle size of nanocarrier in the range of 210 nm to 285 nm low PDI value (0.18). EE and DL were high at 81% and 7.4 mg/100 mg respectively. NP-pRIF showed stable under low temperature (4 °C) and monodisperse delivery system with controlled drug released profile. NP-pRIF showed 2-fold more internalized by *M. tuberculosis* H37RV strain (ATCC 27294) containing macrophage and showed nontoxic J774 A.1 cell line.	[114]
To develop RIF-loaded chitosan nanoparticle dry powder to improve therapeutic approach for alveolar TB.	Pulmonary	Dry Powder Inhaler	Polymeric Nanoparticles	Freeze drying method	Chitosan TPP	J774 cell lines Male Wistar rats	The RIF-loaded chitosan-TPP formulation possess MMAD of 3.3 ± 0.18 µm and the FPF of 33.27 ± 0.87%. RIF-loaded chitosan-TPP formulation had sustained drug release properties. The drug concentration in the lungs were 1.5 and 2.1-fold higher compared to conventional DPI and orally administered RIF solution respectively.	[121]
To develop inhalable formulations of RIF by using supersaturated aqueous solutions.	Pulmonary	Dry powder inhaler	Microparticles	Spray drying Method	NA	NA	The MMAD of the RIF-Aq and RIF-SP formulations were range from 1.4 µm to 1.6 µm with the FPF of higher than 80% and GSD value of 2.9–3.4. RIF formulations showed an high EF of >98 to 69.1 ± 2.3%.	[122]
To develop and evaluate the chitosan microparticles based DPI of RFB.	Pulmonary	Dry powder inhaler	Polymeric microparticles	Spray drying Method	Chitosan TPP Lactose	Female Sprague Dawley rats	RFB-loaded microparticles showed the suitability in deep lungs delivery with the MMAD of <5 µm and FPF of >30% with 1.2 GSD. RFB-loaded microparticles can be internalized by AMs.	[124]
To develop RFB-loaded SLNs for inhaled antitubercular therapy	Pulmonary	Dry powder inhaler	Solid lipid nanoparticles	Lyophilization	GDB/GTT Tween 80	THP-1 cell lines A549 cell lines Calu-3 cell lines	Nanoparticles was in the size range of 121–129 nm, with the PDI range between 0.17–0.20. The RFB-loaded SLNs formulations did not show significant toxicity to respiratory associated cells. The RFB-loaded SLNs formulations can be efficiently taken up by macrophages.	[127]
To develop RFP-loaded PLGA microparticles for TB inhaled therapy.	Pulmonary	Dry powder inhaler	Polymeric microparticles	Spray drying MethodorO/W single emulsion solvent evaporation	PLGA PVA	THP-1 cell lines H549 cell lines	The MMAD of all the PLGA formulations were 2.3–3.1 µm with a span of around 2–3. The PLGA formulations possess a high FPF of 40–57% The RFP-loaded PLGA microparticles can be taken up by macrophages efficiently.	[132]
To develop RFP-loaded proliposomes DPI formulation to treat TB	Pulmonary	Dry Powder Inhaler	Proliposomes	Spray drying Method	HSPC l-leucine Cholesterol Stearyl amine	Albino rats	Size of RFP was around 7.73 µm with the angle of repose between 23–31° and low tapped density of between 0.105–0.677 g/cc indicates good flow properties.	[135]
							MMAD of all formulations was within a range of 1.56–5.26 µm with FPF (<4.6 µm) falling between 60–92%. The RFP concentration in lungs was found to be more than 2-fold higher in all the spray dried formulations compared to pure RFP. The RFP proliposomes formulations showed a 7-fold increase in both mean residence time and drug elimination half-life.	
To compare inhalable crystalline and amorphous dry powder form of RFP by in vitro test	Pulmonary	Dry powder inhaler	NA	Spray drying method	Acetone Methanol	Calu-3 cell lines NR8383 cell lines A549 cell lines	MMAD for both crystalline and amorphous particles fall within the desirable range (1.68 µm and 1.92 µm, respectively) for inhalation. Amorphous formulation exhibited chemically unstable with quinone degradation and color of powder changed occurred. Crystalline and amorphous formulation showed the same MIC as raw RFP and RIF. Solubilized RFP showed five times greater taken up by AMs than RIF.	[167]
To formulate dual antibiotherapy of TB by inhalable LBG microparticles.	Pulmonary	Dry powder inhaler	Microcarriers	Spray drying Method	LBG	THP-1 cell lines A549 cell lines NR8383 cell lines	Microparticle size was 1.15 ± 0.51 µm. Besides, INH and RFB obtained the MMAD equal to 6.2 µm and 5.8 µm. FPF and ED of the formulation was found to be 38% and 92%, respectively. LBG-based microparticles possess a very high macrophages uptake efficiency of 95–100%.	[139]
To characterize matrix embedded formulation for combination spray-dried particles comprising PZA and RIF.	Pulmonary	Dry powder inhaler	Polymeric microparticles	Spray drying Method	HBCD	A549 cell lines	Drug-loaded HBCD formulations showed a good aerosolization properties with MMAD of <5 µm and FPF of >30%.	[141]
To investigate the effects of co-spray drying hygroscopic KNM with hydrophobic RIF in improving the aerosolization of KNM powder for treating respiratory infections.	Pulmonary	Dry Powder Inhaler	NA	Spray drying Method	NA	A549 cell lines Calu-3 cell lines	The combination formulation showed a significant improvement in FPF and yield compared to spray drying KNM alone. The combination formulation obtained the MMAD of 1.4–1.9 µm and the FPF > 77.6%.	[145]
To develop RIF, INH and VPM loaded sodium hyaluronate nanocomposite respirable microparticles to tackle antibiotic resistance mycobacterial pulmonary infections.	Pulmonary	Dry powder inhaler	Microcarriers with nanosize drug	Spray drying Method	Sodium Hyaluronate	HMDM	This combination formulation showed a reduction of 80% bacterial viability, irrespective to the drug-resistance strains at day-3 post infection. The powder of the formulation demonstrated a high FPF of >76% in Turbospin and >90 in RS01. Most of the particles in the combination formulation possess the aerodynamic diameter of <5 µm.	[149]
To develop dry powder formulation combining BDQ with PZA for latent and drug-resistant TB.	Pulmonary	Dry Powder Inhaler	Microcarriers	Spray drying Method	l-leucine	A549 cell lines Calu-3 cell lines	BPL formulation showed a MMAD of 2.9–3.2 µm and FPF of >66%. BPL formulation possess the ability to reduce cytotoxicity of BDQ.	[150]
To develop triple combination dry powder formulation combining BDQ, PZA and MX for the treatment of drug-resistant TB.	Pulmonary	Dry powder inhaler	Microcarriers	Spray drying method	l-leucine	A549 cell lines Calu-3 cell lines	BPML formulation showed a MMAD of <2.5 µm and FPF of >75%. BPML formulation possess the ability to reduce cytotoxicity of BDQ.	[156]
To compare CAF01 co-spray-dried with H56, reconstitution to liquid formulation with the non-spray-dried formulation to induce systemic Th1, Th17 and humoral responses	Pulmonary	Dry powder vaccine	Liposomes	Spray drying method	DDA bromide TDB	Female CB6F1 (BALB/c x C%7BL/6) hybrid mice	Co-spray-dried H56/CAF01 vaccine resulted in preserved antigenic epitopes recognition and adjuvant activity of CAF01. The co-spray dried, reconstituted H56/CAF01 vaccine showed similar in cell mediated and humoral immune responses as the non-spray dried H56/CAF01 formulation.	[158]
To encapsulate MIP and BCG into inhalable alginate particles as DPA to evaluate their immunogenic and protective efficacy in animal model of TB	Pulmonary	Dry powder aerosol	Microcarriers	Spray drying method	Iso-leucine Alginic acid	C57BL/6 mice BALB/c mice	DPA of alginate encapsulated MIP and BCG provided high protection in mice than liquid aerosol with better uptake by BMDCs and co-localized with lysosomes. Alginate encapsulated MIP and BCG DPA showed better presenting of antigen to the T cells as the CCR7 receptors were upregulated in MEAP/BEAP immunized mice. Minimal granulomatous lesions were observed mice immunized with MEAP/BEAP.	[163]
To investigate inhalable dry powder AERAS-402 vaccine in term of physical stability and aerodynamic properties	Pulmonary	Dry powder inhaler	Microcarriers	Spray drying method	Mannitol sucrose β-Cyclodextrin Dextran Leucine PVP	NA	MCTD formulation showed the most thermostability stabilizer with *Tg* value of 97.09 °C, which was the highest among all prepared formulations. Desired aerodynamic characteristics were demonstrated in mannitol-based formulations with narrow size distribution (3.2–3.5 µm). MCTD formulation had higher resistance to water adsorption. MCTD dry powder AERAS-402 showed good stability with 0.12 log loss of virus activity after 5 weeks of storage at 37 °C.	[165]
To formulate inhalable powder vaccine by conjugating Culps1-6 fusion and MPT83 to lipokel for pulmonary delivery	Pulmonary	Dry powder inhaler	NA	Spray drying method	Mannitol	C57BL/6 mice	High sustained influx of neutrophils, myeloid DCs, monocytes and pulmonary macrophage in lung of Culps1-6-Lipokel immunized mice. Protein (Culps1-6 fusion and MPT83)-lipokel conjugates showed increases in frequency of activated DCs in MLN.	[166]
							CD4+ and CD8+ effector T-lymphocytes were increased as well as the induction of antigen-specific, IFN -gamma producing T-lymphocytes was enhanced in proten-lipokel conjugates inmmunized mice. Protein-lipokel conjugates provided significant protection against *M. tuberculosis* infected mice.	
To formulate LEV-proliposomes DPI using porous mannitol to enhance drug delivery to the lungs	Pulmonary	Dry powder inhaler	Proliposomes	Spray drying method	Mannitol Cholesterol from lanolin l-infinity soybean phosphatidyl-choline (SPC)	Wistar rats NR8383 cell lines Calu-3 cells A549 cells line	The MMAD of all the LEV-proliposomes powder were in the ranged of 4.15–4.44 µm with FPF in the ranged of 13–38%. LEV-proliposome powders with 4.15 µm produced the highest ED with 91.3%. LEV-proliposomes exhibited no toxicity on Calu-3, and negligible amounts of inflammatory cytokines and NO were produced by NR8383 cells, which were insufficient to generate secondary inflammation. LEV-proliposome showed good antimycobacterial activity against *Mycobacterium bovis*, *M. tuberculosis* and intracellular *Mycobacterium bovis* in macrophage cells.	[133]
